# Computational Reconstruction of Iron- and Manganese-Responsive Transcriptional Networks in α-Proteobacteria

**DOI:** 10.1371/journal.pcbi.0020163

**Published:** 2006-12-15

**Authors:** Dmitry A Rodionov, Mikhail S Gelfand, Jonathan D Todd, Andrew R. J Curson, Andrew W. B Johnston

**Affiliations:** 1 Institute for Information Transmission Problems, Russian Academy of Sciences, Moscow, Russia; 2 Burnham Institute for Medical Research, La Jolla, California, United States of America; 3 Department of Bioengineering and Bioinformatics, Moscow State University, Moscow, Russia; 4 School of Biological Sciences, University of East Anglia, Norwich, United Kingdom; State University of New York Buffalo, United States of America

## Abstract

We used comparative genomics to investigate the distribution of conserved DNA-binding motifs in the regulatory regions of genes involved in iron and manganese homeostasis in alpha-proteobacteria. Combined with other computational approaches, this allowed us to reconstruct the metal regulatory network in more than three dozen species with available genome sequences. We identified several classes of *cis-*acting regulatory DNA motifs (Irr-boxes or ICEs, RirA-boxes, Iron-Rhodo-boxes, Fur-alpha-boxes, Mur-box or MRS, MntR-box, and IscR-boxes) in regulatory regions of various genes involved in iron and manganese uptake, Fe-S and heme biosynthesis, iron storage, and usage. Despite the different nature of the iron regulons in selected lineages of alpha-proteobacteria, the overall regulatory network is consistent with, and confirmed by, many experimental observations. This study expands the range of genes involved in iron homeostasis and demonstrates considerable interconnection between iron-responsive regulatory systems. The detailed comparative and phylogenetic analyses of the regulatory systems allowed us to propose a theory about the possible evolution of Fe and Mn regulons in alpha-proteobacteria. The main evolutionary event likely occurred in the common ancestor of the Rhizobiales and Rhodobacterales, where the Fur protein switched to regulating manganese transporters (and hence Fur had become Mur). In these lineages, the role of global iron homeostasis was taken by RirA and Irr, two transcriptional regulators that act by sensing the physiological consequence of the metal availability rather than its concentration per se, and thus provide for more flexible regulation.

## Synopsis

The availability of hundreds of complete genomes allows one to use comparative genomics to describe key metabolic processes and regulatory gene networks. Genome context analyses and comparisons of transcription factor binding sites between genomes offer a powerful approach for functional gene annotation. Reconstruction of transcriptional regulatory networks allows for better understanding of cellular processes, which can be substantiated by direct experimentation. Iron homeostasis in bacteria is conferred by the regulation of various iron uptake transporters, iron storage ferritins, and iron-containing enzymes. In high concentrations, iron is poisonous for the cell, so strict control of iron homeostasis is maintained, mostly at the level of transcription by iron-responsive regulators. Despite their general importance, iron regulatory networks in most bacterial species are not well-understood. In this study, Rodionov and colleagues applied comparative genomic approaches to describe the regulatory network formed by genes involved in iron homeostasis in the alpha subclass of proteobacteria, which have extremely versatile lifestyles. These networks are mediated by a set of various DNA motifs (or regulatory signals) that occur in 5′ gene regions and involve at least six different metal-responsive regulators. This study once again shows the power of comparative genomics in the analysis of complex regulatory networks and their evolution.

## Introduction

Iron is an extremely important element in biology. This metal is an integral part of heme and is used as a cofactor in Fe-S proteins. Among the most important cellular functions of iron proteins are protection from oxidative and nitrosative stresses (catalases, peroxidases, oxygenases), nitrogen fixation (nitrogenases), hydrogen production and consumption (hydrogenases), photosynthesis, and methanogenesis [1]. To supply these huge and varied iron needs, bacteria have evolved multiple systems for iron uptake. The ferrous (Fe^2+^) form of iron is acquired by a specific type of transporter, FeoAB [2], whereas ferric iron (Fe^3+^) is taken up by Fbp-type ATP-binding cassette (ABC) transporters [3]. Since ferric iron is extremely insoluble, many organisms produce specific iron-binding agents called siderophores, which solubilize iron, the Fe^3+−^siderophore complex being imported via specific transport systems, usually including an ABC transporter and an energy-transducing system provided by TonB and ExbBD [1,4]. Heme can also act as an iron source for many animal- and plant-associated bacteria, this molecule being imported by dedicated transport systems [5]. To avoid cell damage by excess iron caused by the Fenton-reactive radicals, organisms maintain homeostasis of intracellular Fe by (i) regulating the levels of expression of the genes involved in Fe uptake; (ii) using intracellular storage proteins, such as bacterioferritins; (iii) selective expression of Fe-dependent and Fe-independent enzymes in cells grown, respectively, in Fe-replete and Fe-depleted media [1]; and (iv) detoxification of iron by specific efflux systems [6].

The best-known system of iron homeostasis in bacteria is mediated by the Fur (ferric uptake regulator) transcriptional regulator, which has been extensively studied in various taxonomic groups including γ- and β-proteobacteria, bacilli, and cyanobacteria [7–16]. When Fur interacts with ferrous iron in Fe-replete conditions, it binds avidly to conserved sequences, known as FUR-boxes, and represses the initiation of transcription of its target genes. Global expression analyses in Escherichia coli show that Fur can repress up to 100 genes in iron-rich medium, many of which are directly involved in iron uptake, but others have more tangential links to Fe metabolism [17]. On the other hand, several genes, such as those encoding bacterioferritin and iron-containing enzymes (e.g., fumarase, superoxide dismutase) are positively regulated by Fur through the repression of a small antisense RNA [18].

In contrast, known global iron-responsive repressors in Gram-positive actinobacteria belong to a distinct protein family (represented by IdeR in Mycobacterium tuberculosis and by the closely related DtxR in Corynebacterium diphtheriae), which have no sequence similarity to Fur, although the tertiary structures of the DtxR/IdeR and the Fur proteins do resemble each other [19,20]**.** The DtxR family of metalloregulators also includes the manganese-responsive repressor MntR from enterobacteria, bacilli, and actinobacteria [21–23]. In addition to the global Fe-responsive factors, there are several local regulators, such as the specialized FecI σ-factor in E. coli or the AraC-type transcriptional factor AlcR in *Bordetella,* which regulate genes involved in the uptake of ferric citrate or of siderophores, respectively, and which are components of Fe-responsive regulatory cascades that are mediated by Fur [24,25].

The α-proteobacteria comprise a very widespread, diverse group of organisms that affect many aspects of life on Earth. Some are plant pathogens *(Agrobacterium),* some infect animals (e.g., *Brucella*, *Bartonella*, Rickettsias), some are plant symbionts (e.g., *Rhizobium, Bradyrhizobium*), and many affect environmental parameters, ranging from photosynthesis by *Rhodobacter* to the degradation of xenobiotics by *Novosphingobium*. Also, as shown by the massive sequencing of bacterial metagenomes in the ocean, a huge majority of α-proteobacteria have never been grown, named, or studied [26]. Cosmopolitan oceanic α-proteobacteria from the SAR11 clade (e.g., Pelagibacter spp.) are the most widespread organisms on the planet [27]. Given the key role of iron in processes ranging from photosynthetic reaction centres in *Rhodobacter* and N_2_ fixation in rhizobia to the magnetite crystals of *Magnetospirillum,* it is remarkable how very few direct studies have been done on Fe-responsive gene regulation in α-proteobacteria.

A close homologue of Fur has been identified and studied in the α-proteobacterial rhizobial species Rhizobium leguminosarum and *Sinorhizobium meliloti,* but this “Fur” is not involved in the global regulation of iron uptake. It has a different and more minor role, mediating Mn^2+^-dependent repression of the manganese transporter operon *sitABCD,* and thus was termed “Mur” (manganese uptake regulator) [28–31]. In *Bradyrhizobium japonicum,* a micro-symbiont of soybeans, a Fur homologue is an iron-responsive transcriptional repressor that affects iron uptake in vivo [32] and regulates another iron regulatory gene (*irr*) by direct binding to its upstream region in vitro [33].

Instead of Fur, another protein, called RirA, was identified as being a global iron-responsive transcriptional regulator of iron uptake and metabolism in *Rhizobium* and *Sinorhizobium* [34,35]. Most importantly, RirA represses expression of genes involved in ferrous iron and heme transport, siderophore biosynthesis and transport, and synthesis of Fe-S clusters [34]. Subsequent proteomic studies on R. leguminosarum [36] and transcriptomic analysis in S. meliloti [37] confirmed and extended the wide-ranging effects of RirA on Fe-dependent gene expression. RirA belongs to the large and widespread Rrf2 family of transcription factors, which have no sequence similarity to the Fur- or DtxR-like regulators. The best-characterized members of this Rrf2 family are the Fe-S cluster biogenesis regulator IscR in E. coli [38,39] and the nitrite-responsive regulator NsrR from γ- and β-proteobacteria [40]. In *R. leguminosarum,* strong circumstantial evidence shows that the RirA repressor binds to *cis*-acting motifs at promoters of iron uptake genes [41]. These motifs were named *iron-responsive operators* (IRO) [42].

Another iron-responsive regulator from the Fur superfamily, called Irr, was originally identified in B. japonicum as a repressor of the heme biosynthesis gene *hemB* in iron-limited cells [42,43]. The B. japonicum Irr protein is very unstable in Fe-replete cells. This post-translational instability is mediated by an interaction between heme (whose intracellular concentration is positively correlated with that of the extracellular Fe) and at least two different regions of the B. japonicum Irr protein [44]. One of these regions, the N-terminal heme-recognition motif, is not conserved in the Irr regulators of most species, including *Brucella abortus,* whose Irr has been shown to bind heme in vitro [45]. The B. japonicum Irr protein can bind to a conserved *cis*-acting motif, called ICE (iron control element) [46]. The ICE motifs, with the consensus 5′-TTTAGAA-N_3_-TTCTAAA-3′, were observed upstream of many B. japonicum genes that had a clear link with iron. It has been proposed that, depending on its location within promoter regions, the ICE is involved in either positive or negative control of gene expression [46,47]. Recent microarray studies of the *irr* mutant identified many other Irr targets in B. japonicum and confirmed that Irr is both a positive and a negative effector of iron-dependent gene expression [48]. The Irr ortholog in R. leguminosarum also acts in response to iron availability as an ICE-dependent regulator of transcription of a wide range of genes, including those involved in heme biosynthesis, Fe-S biogenesis, and ferric siderophore uptake [49].

Recently, the experimental findings on iron-controlled gene expression in rhizobia were reviewed by Rudolph et al. [50], who presented the differences and similarities with regard to the operators, regulons, and structure of the iron regulatory proteins RirA, Irr, and Fur/Mur that had been investigated in three species of rhizobia (*B. japonicum, R. leguminosarum,* and S. meliloti).

The work described in this paper extends the study of Fe-responsive gene regulation to many more bacterial lineages and more candidate Fe-regulated genes. The availability of nearly 40 genome sequences for the α-proteobacteria group prompted us to investigate their iron regulatory networks using computational identification of regulatory sites and comparative genomics approaches [40]. Here, we describe the tentative genomic reconstruction of the iron (Fur, Irr, and RirA), manganese (Mur and MntR), and Fe-S biogenesis (IscR) regulons in the sequenced species of α-proteobacteria and show significant variability and connectivity in these regulatory networks. For each genome, we report computational identification of several classes of *cis-*acting iron regulatory DNA motifs (including ICE-, IRO-, and Fur-boxes) in the 5′-regions of most genes involved in iron uptake and storage, and usage. In several lineages and species of α-proteobacteria, we observed lineage-specific members of iron regulons, overlap between regulons, and potential regulatory cascades involving different iron regulators. Finally, we discuss potential evolutionary scenarios for this unique regulatory network.

## Results/Discussion

### Phylogenetic Analysis of Iron/Manganese Regulators in α-Proteobacteria

**Figure 1:**
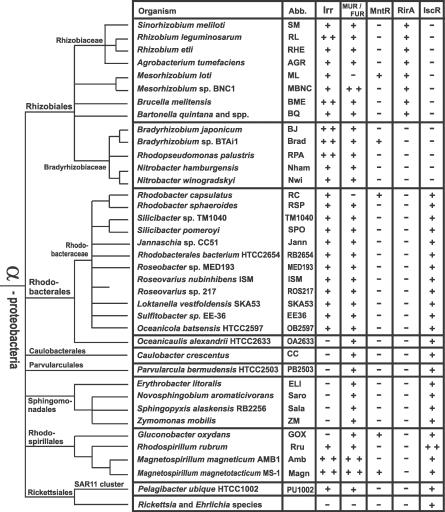
Distribution of Irr, Fur/Mur, MntR, RirA, and IscR Regulators in α-Proteobacteria Genome abbreviations are given in the second column. The conventional taxonomic tree of α-proteobacteria shown on the left of the table was adapted from the NCBI Taxonomy Homepage (http://www.ncbi.nlm.nih.gov/Taxonomy/taxonomyhome.html) and is in agreement with the tree of α-proteobacteria based on 16S rRNA sequences [64]. Double plus (“++”) denotes the presence of two genes encoding copies of a given transcription factor.

We searched the genomes of α-proteobacteria available in the Genbank database (http://www.ncbi.nlm.nih.gov/Genbank, as of August 2006) for homologs of each of five known wide-ranging regulators (RirA, Irr, Mur, Fur, and MntR) that respond to Fe and/or Mn availability. In addition, we considered the phyletic distribution of orthologs of the IscR regulator, which controls the Fe-S biogenesis genes in E. coli [39], since the latter genes are also frequent members of iron regulons [49,51]. The occurrence of these six classes of transcriptional regulators among the analyzed bacterial lineages is summarized in Figure 1.

#### Fur/Mur.

**Figure 2:**
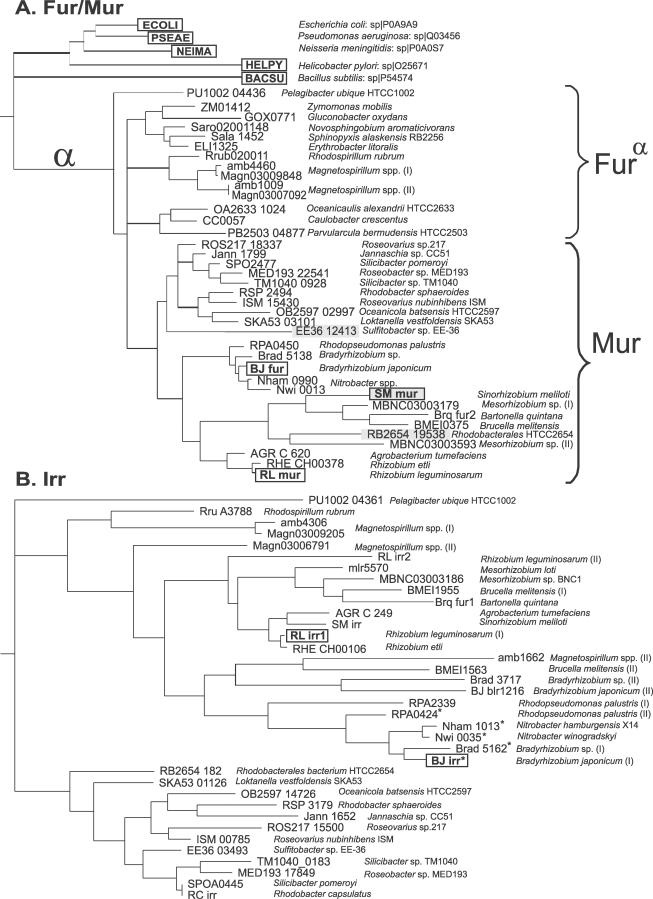
Phylogenetic Tree of α-Proteobacterial Regulators from the Fur Superfamily (A) Fur/Mur, (B) Irr. Experimentally characterized regulatory proteins are in bold and boxed. Positional clustering (i.e., close linkage) of the *mur* genes with the target manganese uptake *sit* operons is shown by background grey. Irr proteins with the heme regulatory motif (see text) at their N-termini are marked with asterisks. Functional annotation of the “mur” and “fur^α^” regulators is based on the genomic analysis of their candidate regulatory motifs that occur in 5′ regions of either manganese or iron uptake genes, respectively. Possible role of the B. japonicum regulator Fur in the control of the manganese uptake gene *mntH* was predicted in this study and is not yet proved.

Orthologs of the conventional Fur regulator are present in nearly all α-proteobacteria, forming a compact branch on the phylogenetic tree of the Fur superfamily (Figure 2A). The “Fur” protein in at least two rhizobia species is a “Mur,” manganese uptake regulator. The exceptions, which lack any such Fur-like proteins, are Rhodobacter capsulatus and Mesorhizobium loti and pathogens from the Rickettsiales order. We noticed that in both *Mesorhizobium* and *Rhodobacter* genera, one each of the species whose genome had been sequenced contained the *fur*-like gene, but the other did not (Figure 1). However, and unusually, these two strains have orthologs of the Mn-responsive repressor MntR from the DtxR family (see the “MntR regulon” section below).

The characteristic Fe-binding motif “His-His-Glu-His” that is present in Fur proteins from γ-proteobacteria is conserved in all Fur/Mur orthologs in α-proteobacteria [19]. Among these Fur/Mur proteins, only three have been characterized experimentally: the Mn-responsive regulators of the Mn^2+^ uptake operon in S. meliloti and *R. leguminosarum,* and the Fe-responsive regulator of the *irr* gene in B. japonicum [23–28]. Some *mur* genes are adjacent on the chromosome to the manganese uptake *sit* operons. Functional analysis of genes preceded by the candidate binding sites of Fur/Mur regulators (see next section) allowed us to tentatively distinguish regulators of iron homeostasis (Fur) from the regulators of manganese uptake (Mur).

#### Irr.

This highly unusual set of the iron-responsive regulators is found only in some lineages of the α-proteobacteria and forms a distinct branch within the Fur superfamily, and which differs significantly from the Fur/Mur proteins (Figure 2B). The N-terminal DNA-binding domains in the Irr and Fur/Mur proteins are more similar to each other than are their C-terminal dimerization domains. The Fe-binding motif of Fur proteins is not conserved in Irr proteins although two or three His residues are present in the corresponding regions of these proteins. Irr orthologs are present in all analyzed genomes from the Rhizobiales and Rhodobacterales orders of α-proteobacteria, as well as in the *Rhodospirillum* and *Magnetospirillum* species (Figure 1). Some of these genomes (*Bradyrhizobium, Rhodopseudomonas, Brucella, R. leguminosarum,* and *Magentospirillum* species) encode two Irr paralogs that do not show strong pairwise sequence similarity within the sequenced strains. In addition, an Irr ortholog with less sequence similarity to the others was found in *Pelagibacter ubique,* a cosmopolitan oceanic bacterium from the SAR11 clade of α-proteobacteria, which is rather distantly related to the other Irr-containing lineages [27]. As mentioned, at least one Irr protein in bacteria from each strain in the Bradyrhizobiaceae group contains a heme-recognition motif, but this motif is missing from all other Irr proteins (Figure 2B).

#### RirA.

**Figure 3:**
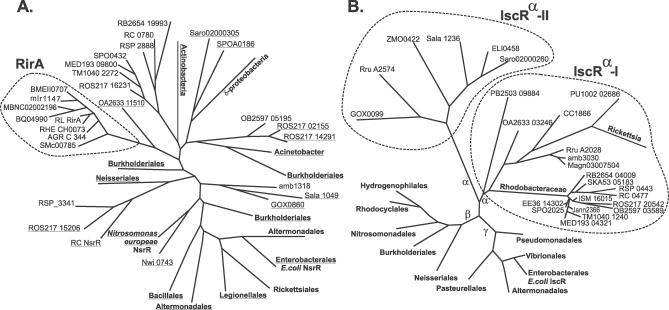
Phylogenetic Tree of the α-proteobacterial Regulators from the Rrf2 Superfamily (A) RirA/NsrR, (B) IscR. Genome abbreviations are listed in Figure 1. *nsrR/rirA* homologs positionally linked with the nitrosative stress response genes are underlined.

The iron-responsive regulator RirA is a member of the Rrf2 superfamily of transcriptional regulators and belongs to the subfamily of the NsrR-like regulators (Figure 3A) that regulate various genes involved in the nitrogen oxide metabolism in Gram-positive and Gram-negative bacteria [40]. The R. leguminosarum RirA protein has orthologs (∼70% identity) in the genomes of other sequenced genera of the Rhizobiaceae group, as well as in *Brucella, Bartonella,* and *Mesorhozobium* species (Figure 1). However, RirA is absent in the genomes of other Rhizobiales species from the Bradyrhizobiaceae group including *B. japonicum, Rhodopseudomonas palustris,* and *Nitrobacter* species.

Many regulatory proteins are encoded by genes that are closely linked to at least some of the genes that they regulate [52]. Thus we noted positional linkage of the *rirA* gene with iron uptake or storage genes in five genomes (*S. meliloti, Agrobacterium, Brucella,* and *Mesorhizobium* species). Outside of the Rhizobiales group, *rirA* homologs are often adjacent to nitrosative stress genes (Figure 3A) and are supposed to regulate nitrogen oxide metabolism genes, similarly to the nitrite-responsive repressor NsrR [40].

#### IscR.

The IscR regulator of the Fe-S cluster biogenesis genes also belongs to the Rrf2 superfamily [39]. IscR orthologs are widespread in proteobacteria (Figure 3B). Among α-proteobacteria, they were found in the Rhodobacterales, Rhodospirillales, Sphingomonadales, Rickettsiales, and Caulobacterales orders, where they are always located immediately upstream of the Fe-S synthesis *suf* genes. Although the *suf* genes are ubiquitous among α-proteobacteria, there is no cognate *iscR* regulator in the Rhizobiales order (Figure 1). In contrast, regulation of the *suf* genes in Rhizobiales is mediated by the wide-ranging iron regulators RirA and Irr [36].

### Identification of Regulatory Motifs and Reconstruction of Regulons

We used an ab initio bioinformatic approach to identify conserved sequences in the regulatory regions of potentially Fe-regulated operons in the α-proteobacteria. To do this, we applied the DNA motif detection procedure to a training set of upstream regions of various iron uptake genes from the bacteria whose genomes encode the specific, cognate transcriptional factor. The initial training set of 5′ gene regions and the procedure used to predict the regulon members were modified in each case, depending on the nature of the available experimental data for the particular regulon and/or by analogy with the respective regulatory systems in other classes of bacteria (see Materials and Methods for details). The predicted regulatory motifs for the transcriptional factors Mur, Fur^α^, RirA, Irr, IscR, and MntR in α-proteobacteria, the position of sites relative to the proximal downstream genes, names, and annotation of the candidate regulon members are listed in Tables S1–S7.

#### RirA regulon.

**Figure 4:**
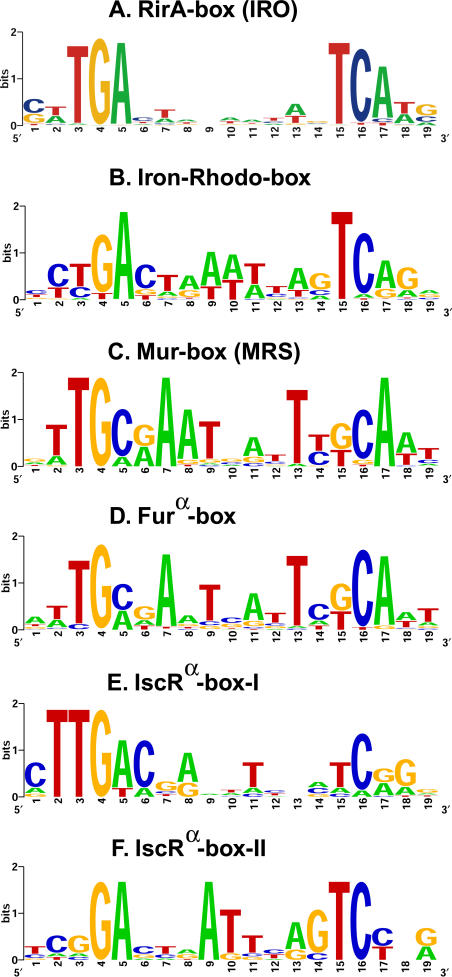
Sequence Logos for the Predicted Regulatory Sites in α-Proteobacteria (A) RirA-box (IRO) in eight species from the Rhizobiales order (four Rhizobiaceae, two *Mesorhizobium* species, *Brucella,* and *Bartonella*). (B) Iron-Rhodo-box in the Rhodobacteraceae. (C) Mur-box (MRS) in the Rhodobacteraceae/Rhizobiales. (D) Fur^α^-box in other α-proteobacteria species. (E) IscR^α^-I motif in the Rhodobacterales, the Rickettsia, *Pelagibacter, Oceanicaulis, Caulobacter, Parvularcula, Rhodospirillum,* and *Magnetospirillum* species. (F) IscR^α^-II motif in the Sphingomonadales and *Gluconobacter* species.

Analysis of upstream regions of candidate iron uptake genes in α-proteobacteria possessing a close RirA ortholog (the Rhizobiales species except the Bradyrhizobiaceae group; Figure 1) yielded a conserved palindromic motif with the consensus 5′-TGA-(N_9_)-TCA-3′ (Figure 4A). The newly proposed RirA recognition motif, named RirA-box, overlaps and slightly differs from the previously identified IRO motif in the promoter regions of some genes in R. leguminosarum that were shown to be repressed by RirA in Fe-replete conditions [35]. The newly constructed recognition profile was used to scan the genomes of Rhizobiales for additional candidate RirA-boxes, allowing us to tentatively identify many additional candidate targets of RirA (Table S1). For all these species except, notably, the Bradyrhizobiaceae group, we observed many highly significant matches in the upstream regions of genes involved in iron metabolism. The largest class of genes with candidate RirA-boxes in upstream regions is involved in iron uptake and storage.

#### RirA-box verification.

**Figure 5:**
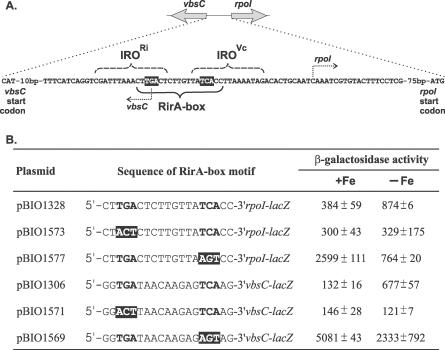
Validation of the Predicted RirA Recognition Motif in R. leguminosarum by Site-Directed Mutagenesis (A) RirA-box in the common intergenic region of the RirA-regulated *vbsC* and *rpoI* genes in R. leguminosarum. The sequence of this region is shown where the transcription start sites are in bold and marked by arrows. The previously identified IRO-boxes for *vbsC* and *rpoI* [41] are under the dashed line brackets. The highly conserved “TGA” and “TCA” in the newly described RirA-box are highlighted. (B) Effect of mutating the conserved regions of the RirA-boxes on Fe-responsive expression of *rpoI-lacZ* and *vbsC-lacZ* transcriptional fusions. The previously described [41] plasmids pBIO1328 and pBIO1306 are based on the wide host-range promoter probe plasmid pMP220 [80] and contain the promoter and regulatory regions of *rpoI* and *vbsC,* respectively, fused to its promoter-less *lacZ* gene. In addition, four new sets of mutant derivatives were made, in which the conserved “TGA” and “TCA” sequences of the RirA-box were substituted, using methods described by Yeoman et al. (2004). Mutant derivatives of pBIO1328 and pBIO1306 with substitutions of the conserved TGA and TCA sequences of the RirA-box were made using the Stratagene ExSite PCR-based Site-directed Mutagenesis kit, with each of these two plasmids being used as template and a suitable oligonucleotide as the mutagenic primer. The mutated forms are shown with dark backgrounds. Each of the six plasmids was individually mobilized into wild type *R. leguminosarum.* Transconjugants were grown in Fe-replete and Fe-depleted medium and assayed in triplicate for β-galactosidase activity as in Wexler et al. [81].

To confirm the importance of this novel iron regulatory motif, we conducted site-directed mutagenesis of the candidate RirA-boxes in the promoter regions of two divergently transcribed genes (*vbsC* and *rpoI*) in R. leguminosarum (Figure 5). Each of the most highly conserved parts of this RirA-box was separately mutated, and the Fe-responsive expression of *rpoI-lacZ* and *vbsC-lacZ* transcriptional fusions was measured. In both cases, the substitution of the TCA with AGT caused hyperexpression in the presence of Fe, with no RirA-mediated repression being seen in the “+Fe” conditions. In contrast, the TGA to ACT substitution decreased the overall level of expression of *rpoI* and *vbsC* and abolished Fe-determined repression. Therefore, this region might actually form part of the promoter, as well as having a role in the Fe-responsive gene regulation. Thus, the conserved triplets within the newly identified RirA-box are indeed involved in the RirA-dependent repression in the presence of Fe.

#### Iron regulon in Rhodobacteraceae.

In the absence of any experimental data about iron regulation in this group of α-proteobacteria, we attempted to reconstruct their possible iron regulons by applying the motif detection procedure to the set of 5′ regions of candidate iron uptake genes. This resulted in the identification of a highly conserved 19-bp palindromic signal, which we term the Iron-Rhodo-box, which occurs in upstream regions of most iron uptake and storage genes in all 12 of the available genome sequences of the Rhodobacteraceae group (Table S2). The candidate iron regulatory DNA motif in the Rhodobacteraceae is similar to the RirA-box motif in the Rhizobiales (Figure 4A and 4B) and has some resemblance to the known IscR-binding motif from γ-proteobacteria (5′-ctTGActaanttacTCAgg-3′) [38]. This intriguing similarity is discussed in more detail in the last section.

#### Irr regulon.

**Figure 6:**
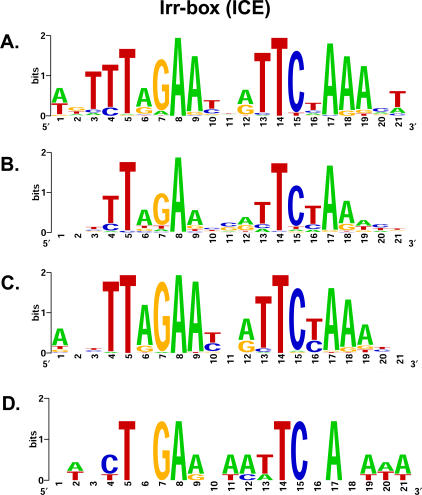
Sequence Logos for the Predicted Irr Recognition Motifs (ICEs) in Various Groups of α-Proteobacteria (A) Rhizobiaceae plus *Mesorhizobium, Brucella,* and *Bartonella* species. (B) Bradyrhizobiaceae group. (C) Rhodobacteraceae group. (D) Rhodospirillales group.

Since the RirA regulon is absent in the Bradyrhizobiaceae, we performed a search for any conserved *cis-*acting regulatory sequences in the upstream regions of genes involved in the iron homeostasis in the four sequenced genomes from this lineage. This identified a conserved motif with palindromic symmetry and the consensus sequence 5′-TTTRGAAYNRTTCYAAA-3′ (Figure 6B). Not surprisingly, this DNA motif is similar to the ICE initially described in B. japonicum by Nienaber et al. [47] and recently confirmed to be a target of the Irr regulator [46]. We noted that this Irr regulatory motif is shared by most B. japonicum iron uptake and storage genes, as well as by other genes involved in the iron metabolism such as *suf, hemA,* and many operons encoding iron-containing enzymes *(acnA, ccm, cyc, fumA, fdh, fdx, hup, ior, katG, nuo, sdh,* and *bll2737-bll2736)*.

To analyze the Irr regulon further, we used the constructed recognition profile to scan the genomes of other α-proteobacteria. Candidate Irr-binding sites were identified in the genomes of all α-proteobacteria from the Rhizobiales and Rhodobacterales orders (Table S3). Several interesting exceptions to this notable phylogenetic distribution of Irr/ICE are *Pelagibacter ubique, Rhodospirillum rubrum,* and *Magnetospirillum* species, whose genomes have only one or two candidate ICE sites. The minimal Irr regulons in these species are predicted to include only either the di-heme cytochrome c peroxidase or a rubrerythrin-like protein constituting a non-heme iron-binding domain. Apart from the above-mentioned species and lineages of α-proteobacteria, Irr regulators and ICE recognition motifs were not found in other bacterial genomes.

**Figure 7:**
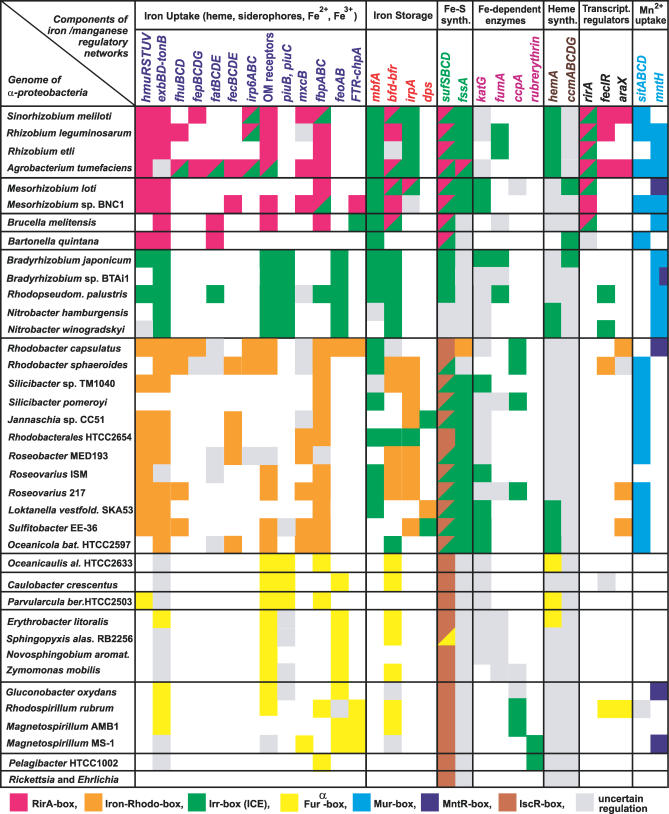
Occurrence of Candidate Regulatory Elements and Genes Involved in the Iron and Manganese Homeostasis in α-Proteobacteria Only conserved members of the predicted Fe/Mn regulons are shown. Genes are arranged by their functional role. Genomes are arranged by taxonomic lineages. When the gene is present in the genome, the background colors denote the presence of the specific recognition motif in its upstream region. If the gene is preceded by two different regulatory motifs, it is shown by a diagonally separated bicolor square. Differential regulation of two *mntH* paralogs in Bradyrhizobium sp. BTAi1 by Fur/Mur and MntR regulons is shown by vertically separated bicolor square. Genes without any candidate iron or manganese regulatory motifs described in this study are indicated by grey background color. Empty crossings denote the absence of an orthologous gene in the genome.

Compared with the Bradyrhizobiaceae, the predicted Irr regulons of other Irr-containing species have many fewer target genes, most of which are involved in iron storage (bacterioferritins) and usage (Fe-S and heme biosynthesis, and some iron-containing enzymes) (Figure 7). Therefore, there is strong correspondence between the presence of Irr-binding motifs (ICE) and at least one *irr* gene in the genome of any given species. Despite the difference in the Irr regulon content, the consensus sequences of ICE motifs (Figure 6) are well-conserved in various lineages of α-proteobacteria.

#### Mur regulon.

In an attempt to describe this manganese regulon in α-proteobacteria, we applied the motif detection procedure to the upstream regions of candidate Mn^2+^ uptake operons, chosen by their homology to known manganese transporters. This identified a 19-bp palindromic motif (Figure 4C; Table S4) that coincides with the Mur-responsive sequences (MRSs) that were shown in DNase I protection assays to be the Mur-binding site of *R. leguminosarum sitABCD* [53]. Two different manganese transport systems, ABC-type *sitABCD* and NRAMP-type *mntH,* were found to be associated with the identified candidate MRS sequences in most α-proteobacteria from the Rhizobiales and Rhodobacterales orders (Figure 7). Three species (R. leguminosarum, Mesorhizobium sp., and *Agrobacterium tumefaciens)* have both *sitABCD* and *mntH* genes, and the upstream regions of both sets of genes contain candidate Mur-responsive sites. In *S. meliloti, Sulfitobacter,* and *Rhodobacterales* species, their *mur* and *sitABCD* genes are closely linked, supporting the hypothesis that the MRS motif is also a target of Mur in the Rhodobacteraceae group.

An interesting exception was found in two Rhodobacterales species, *Roseovarius nubinhibens* ISM and Oceanicola granulosus HTCC2516, which have Mur orthologs but lack any known Mn uptake system, since they have neither MntH nor SitABCD transporters. In both these marine bacteria, an MRS site was found upstream of a conserved gene named *mntX* (the corresponding genomic identificators are *ISM_02005* and *OG2516_13601*), which encodes a predicted integral membrane protein with unknown function. Thus, our analysis may have identified a candidate for another, hitherto unknown, Mn^2+^ transporter.

We also noted weaker MRS-like sequences sites upstream of the iron regulatory gene *irr* in Bradyrhizobiaceae and some Rhodobacterales. In *B. japonicum,* a candidate MRS within the *irr* promoter region coincides with the experimentally defined Fur-binding site [43]. Furthermore, one of the *irr* paralogs in B. melitensis is preceded by a strong-candidate MRS site. Thus, at least in some α-proteobacteria, Fur/Mur's may have a dual role in the control of Mn^2+^ uptake genes and the iron regulatory *irr* genes.

#### MntR regulon.

**Figure 8:**
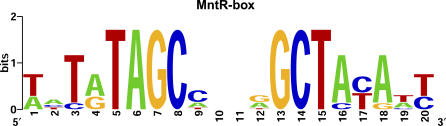
Sequence Logos for the Predicted MntR Recognition Motifs in α-Proteobacteria

The genomes of R. capsulatus and M. loti lack the *fur/mur* gene but do contain a copy of the *mntR* gene, located next to *mntH*. This suggests that, in contrast to many other α-proteobacteria with Mur, the uptake of Mn^2+^ in these two species is regulated by MntR, an ortholog of the Mn^2+^-sensing transcriptional regulator from E. coli and B. subtilis [22,23]. By applying the signal detection procedure to 5′ regions of the *mntH* genes from the MntR-containing genomes of α-proteobacteria, we identified a 20-bp palindromic motif (Figure 8; Table S5), which is similar to the consensus MntR-binding site in enterobacteria (5′-AACATAGCnnnnGCTATGTT-3′) [23]. The presence of *mntR* in the genome of Mesorhizobium sp. BNC1 is enigmatic since the only candidate MntR-binding site was found upstream of *mntR* itself, suggesting its autoregulation, whereas Mn^2+^ uptake transporters in this species (*mntH* and *sit*) are likely under the control of Mur. Interestingly, the Bradyrhizobium sp. strain BTAi1 has two *mntH* paralogs, one of which is a candidate member of the Mur/Fur regulon and has an MRS-like sequence, and the other one of which is preceded by a strong candidate MntR-binding site.

#### Fur^α^ regulon.

We attempted to describe the iron regulons in α-proteobacteria outside of the Rhizobiales and Rhodobacteraceae groups, which have no Irr and RirA regulons but possess Fur/Mur-like proteins (Figure 1). To do this, we performed similarity searches to identify candidate iron uptake genes, and used their upstream regions as training sets for motif detection. This analysis was performed separately for each species from the Rhodospirillales and Sphingomonadales subgroups, as well as for *Caulobacter crescentus, Parvularcula bermudensis* HTCC2503, and Oceanicaulis alexandrii HTCC2633. This resulted in the identification of a set of closely related regulatory motifs (Figure S1) with a common palindromic consensus, which we term the Fur^α^-box. The proposed Fur^α^ recognition motif is similar to the previously defined MRS motif in other α-proteobacteria (Figure 4C and 4D), and has some resemblance to known Fur-binding motifs from various α-proteobacteria and B. subtilis (5′-AATGATAATnATTATCATT-3′) [14–16].

Scanning the genomes with the constructed recognition profiles allowed us to tentatively predict the content of Fur^α ^regulons in the α-proteobacteria lineages that lack both the RirA and Irr regulons (Table S6). The most abundant members of these predicted Fur^α^ regulons are TonB-dependent outer membrane iron receptors (OMP), the ferrous iron transporters *feoAB,* and the ferric iron transporters *fbpABC* and *ftr*/*chpA*. Other candidate iron uptake genes, including *piuB, piuC,* and *exbBD-tonB,* as well as the hemin ABC transporter *hmu,* are predicted to be members of the Fur^α^regulons in at least some species. Also, in some α-proteobacteria, candidate Fur^α^ sites were identified upstream of genes for iron storage (bacterioferritin *bfr*), heme biosynthesis *(hemA),* Fe-S biogenesis *(suf),* and iron-utilizing enzymes *(sdh, nuo).* Finally, we noted an interesting extension of the Fur^α^ regulon in both *Magnetospirillum* species, where, in addition to multiple iron uptake genes, candidate Fur^α^ sites were observed upstream of some genes related to its unusual magnetotactic phenotype, in particular, *mms6, mms7,* and *mms13,* that encode magnetosome membrane proteins. These findings suggest that in addition to its predominant role in regulation of iron uptake, Fur^α^ has an extended role in controlling iron homeostasis in these α-proteobacteria (Figure 7).

Lastly, Pelagibacter ubique is predicted to have only one Fur^α^-regulated operon, encoding the ferric cation ABC transporter Fbp. All other known iron transport and storage genes are missing from its very small genome.

#### IscR regulon.

Expression of the Fe-S cluster biogenesis operons *isc* and *suf* in α-proteobacteria is repressed by the Fe-S cluster–containing transcription factor IscR [39] and the latter operon is also negatively regulated by Fur [51]. The *suf* gene loci in the Rhizobiales genomes are the predicted (and in the case of *R. leguminosarum,* validated) members of the RirA and Irr regulons. Unusually, these genomes lack an ortholog of the IscR repressor. However, the chromosomal clusters of *suf* genes in all other γ-proteobacteria, including obligate pathogenic Rickettsia, include an *iscR* homolog, allowing us to propose that the IscR-dependent mechanism of regulation is conserved in these species.

On the phylogenetic tree of the IscR subfamily there are two separate branches of α-proteobacterial IscR-like proteins (Figure 3B). The IscR^α^-I group is most similar to IscR proteins from γ-and β-proteobacteria, and the respective *suf* loci are preceded by a 19-bp palindromic motif that resembles the IscR-binding motif from α-proteobacteria [39] (Table S7; Figure 4E). The IscR^α^-II group, seen in four Sphingomonadales species and *G. oxydans,* is quite diverged from the IscR^α^-I group, and the predicted DNA recognition motif for the second group has only limited similarity to the IscR^α^-I motif (Figure 4E and 4F).

### Functional Content of Candidate Iron Regulons

The identification of candidate iron regulatory motifs in the genomes of α-proteobacteria allowed us to reconstruct their likely iron regulons (Figure 6). In spite of the variety of regulatory systems in the different taxonomic groups, the structural genes that constitute the core of the iron regulons in α-proteobacteria, and other well-characterized bacteria such as E. coli and *B. subtilis,* almost completely coincide. In other words, if “*geneX”* is regulated in response to Fe in (e.g.) *E. coli,* then the “*geneX”* ortholog is also Fe-regulated in the α-proteobacteria, even though the regulatory protein may be very different. These genes include most of those involved in various aspects of iron metabolism, including iron uptake transporters, iron storage ferritins, and some systems that use iron as a cofactor. The comparative analysis of the iron regulons also allowed us to identify several new components that are likely implicated in iron homeostasis in α-proteobacteria.

#### Iron uptake systems.

The heme utilization *hmu* clusters that encode components of the TonB-dependent transport system, and hemin-degrading proteins, are among the most conserved members of the iron regulons. Depending on the particular species, candidate ICE-box, RirA-box, Iron-Rhodo-box, or Fur^α^-box motifs are present in the regulatory regions of most *hmu* operons (Figure S2). Orthologs of the ferrisiderophore ABC transporters *fhu, fat, fep, fec,* and *irp6* are less common in α-proteobacteria, being found only in some Rhizobiales and Rhodobacterales species. Various TonB-dependent OMPs for ferrisiderophores, which are accompanied by the *exbBD-tonB* genes, are the most numerous representatives of the iron regulons in many α-proteobacteria. The candidate iron regulons also include the majority of the Fbp-type ferric cation ABC transporters and the FeoAB ferrous iron transport systems, which are widely distributed in α-proteobacteria. A putative iron transport operon encoding a homolog of the ferric iron transporter FTR1 from yeast [54], a ferredoxin-like protein, and a periplasmic metal-binding protein ChpA [55] are present only in some species, and are predicted to be regulated by either a RirA-box (Mesorhizobium sp.), an Iron-Rhodo-box (R. capsulatus), an ICE-box (B. melitensis), or a Fur^α^-box (*Rhodospirillum* and *Magnetospirillum* species).

#### Iron storage ferritins.

Bacterioferritin *bfr* and ferredoxin *bfd* (which are nearly always adjacent to each other) are predicted to be controlled by cognate iron regulatory elements in most α-proteobacteria. Another conserved member of the candidate iron regulons, the hypothetical gene *irpA,* was often found to be adjacent to the *bfd-bfr* genes, suggesting that its product could be also involved in iron storage. In agreement with this, the *irpA* gene in a cyanobacterium, Synechococcus sp., is induced under Fe deficiency and is involved in Fe-limited growth [56]. Another highly conserved and rather unusual member of the Irr regulon, which may play a role in iron storage, is encoded by the *mbfA* gene. In R. leguminosarum and *B. japonicum, mbfA* was shown to be repressed directly by Irr in low-iron conditions [46,49]. MbfA has a ferritin-like N-terminal domain and a C-terminal domain with four predicted transmembrane segments that belong to the PF02915 and PF01988 protein families, respectively, according to the PFAM database. The exact function of MbfA is unknown but its domain composition suggests that it may function as a membrane-bound ferritin. Finally, the predicted iron regulons in three Rhodobacteraceae include the *dps* gene, encoding a ferritin-like protein that protects cells from oxidative stress by sequestering iron and limiting Fenton-catalyzed oxy-radical formation [57].

#### Fe-S cluster biogenesis.

The *suf* operon is predicted to be a conserved member of the Irr regulon in most Rhizobiales/Rhodobacterales, as this had been confirmed experimentally in the case of R. leguminosarum [36]. It is also an “optional member” of the RirA regulon in the Rhizobiaceae group, *Brucella* and *Bartonella* species, where it can be regulated by both Irr and RirA or by Irr alone. The cognate Fe-S repressor IscR likely controls the *suf* genes in all α-proteobacteria except the Rhizobiales order, which lacks this regulator. Another gene, *fssA* (iron sulfur scaffold), likely to be involved in the formation and maturation of Fe-S clusters in enzymes, was identified as a conserved member of the Irr regulon in the Rhizobiaceae and Rhodobacteraceae groups, and had been shown experimentally to be regulated by IrrA in R. leguminosarum [49].

#### Heme biosynthesis.

The *hem* genes are present in almost all studied α-proteobacteria [58], but only some of them belong to the predicted iron regulons. The *hemA* gene, which encodes the first dedicated step in the heme synthesis, is often preceded by a candidate ICE motif (the Rhizobiaceae, *Nitrobacter, Sulfitobacter, Loktanella,* and *Oceanicola* species) or a Fur^α^-box (*Oceanicaulis, Erythrobacter,* and *Sphingopyxis* species), whereas *hemH* and *hemN,* which encode enzymes that act at a later stage in the biosynthetic pathway, are rare members of the candidate iron regulons in α-proteobacteria. Though Irr was first identified in B. japonicum as a regulator of *hemB,* rather ironically this heme synthesis gene is not preceded by an ICE site, and the mechanism of its iron control is still unknown [46].

#### Iron-containing enzymes.

Several genes encoding iron-containing enzymes (e.g., *sdh, acnA, fumA, sodB*) are positively regulated in iron-replete conditions by Fur in E. coli through repression of a small antisense RNA [18]. In the Rhizobiales/Rhodobacterales, we identified many genes encoding iron-containing enzymes that are preceded by candidate ICE sites, suggesting that Irr represses these genes at low iron concentrations. These included the heme-containing catalase/peroxidase (*katG*) and cytochrome c peroxidase (*ccpA*), the Fe-S–containing fumarate hydratase (*fumA*), succinate dehydrogenase (*sdh*), NADH ubiquinone oxidoreductase (*nuo*), and some other cytochromes (marked in green text in Table S3). We noted that such Irr-regulated genes are more common in Bradyrhizobiaceae than in other Irr-containing lineages. Some of the orthologs of these proteins in *Oceanicaulis, Parvularcula,* and *Magnetospirillum* species are preceded by candidate Fur^α^ sites, but the mode of the predicted iron regulation of these genes is unknown.

#### Alternative isozymes.

We also observed several examples of another regulatory strategy for iron metabolism. This occurs when an alternative, iron-independent enzyme is negatively regulated by high iron concentrations, as reported for an alternative Cu-containing superoxide dismutase in γ-proteobacteria [59–61]. From our observations here, we tentatively predict that RirA represses expression of the iron-independent fumarate hydratase FumC in *R. leguminosarum, R. etli,* and B. melitensis but not in other α-proteobacteria. Such a regulatory strategy fits nicely with our observation that these three species contain two forms of fumarate hydratase, one of which (FumA) has an Fe-S cluster and the other (FumC) lacks Fe, whereas other rhizobia with the RirA regulon lack an iron-containing isozyme.

#### Transcription factors.

The RirA regulon in S. meliloti includes the AraC-like transcriptional activator RhrA, which controls the rhizobactin synthesis operon [62]. Among other α-proteobacteria, hypothetical AraC-like genes, named *araX,* were predicted to be regulated by iron regulatory motifs in *Agrobacterium, Sulfitobacter, Roseovarius* species, and R. capsulatus. Indeed, the latter has four *araX* paralogs, each of which is located next to ferrisiderophore utilization genes (Table S2). We therefore propose similar regulatory cascades in at least some α-proteobacteria, in which RirA controls the expression of an AraC-type activator, which in turn regulates its cognate ferrisiderophore utilization operon.

Another regulatory system identified in α-proteobacteria, and which might be involved in additional control of iron-regulated genes, is homologous to the FecIR system from enterobacteria. In *E. coli,* the FecR protein serves as a signal receiver in the periplasm and as a signal transmitter across the cytoplasmic membrane to the FecI sigma factor, which, when activated, binds to the RNA polymerase and specifically initiates transcription of the *fecABCDE* ferric citrate transport operon [24]. Transcription of *fecIR* is negatively regulated by Fe^2+^-Fur in E. coli. Here we identified several *fecIR*-like gene loci preceded by either candidate RirA-boxes in some Rhizobiaceae, or by predicted Irr-boxes in some Bradyrhizobiaceae, suggesting the control of their expression by iron concentrations. Finally, the predicted Fur^α^ regulon in Rhodospirillum rubrum includes four *fecIR* homologs (all of which are adjacent to TonB-dependent receptor OMP genes), and three *araX* genes encoding hypothetical AraC-like transcription factors (Table S6).

### Predicted Regulatory Networks versus Expression Microarrays

#### Sinorhizobium meliloti.

As revealed in microarrays, the expression of most S. meliloti genes that have candidate RirA-boxes in their regulatory regions is significantly induced under iron-limiting conditions and in the *rirA* mutant [37]. These include the rhizobactin biosynthesis and uptake genes, the heme acquisition *hmu* gene locus and the hemin receptor *shmR,* various ferrisiderophore ABC transporters and outer membrane receptors, the *exbBD* components of TonB-dependent iron transporters, and the ferric cation ABC transporter *fbp* (highlighted in blue in Table S1). S. meliloti genes involved in the Fe-S cluster synthesis *(suf)* are predicted members of both RirA and Irr regulons, and these genes were differentially expressed in the *rirA* mutant but not under iron-limiting conditions [37]. However, this large-scale transcriptomic study did not reveal some of the iron uptake genes that we found to be associated with candidate RirA-boxes in S. meliloti genome (*hmuR, fhuA, fecIR,* and *rhtX2-viuA*). And, vice versa, many genes that are differentially expressed in the *rirA* mutant are not preceded by significant RirA-box sequences. These apparent discrepancies may indicate that the deregulated iron uptake in the *rirA* mutant might cause secondary transcriptome changes; for example, by altering the intracellular Fe concentrations. Also, RirA may affect gene expression via regulatory cascades that include other transcription factors (e.g., RhrA, FecIR, AraX).

#### Bradyrhizobium japonicum.

A genome-wide transcriptomic survey in this organism revealed sets of iron-induced and iron-repressed genes, many of which were found to contain candidate Irr binding sites (ICE) in their promoter regions [46]. Another microarray expression analysis identified multiple genes that are either downregulated or upregulated in the *irr* mutant strain, or in iron limitation relative to iron-replete cells [48]. In Table 1, we compared these pan-genomic expression studies with the current reconstruction of the Irr regulon presented here. We noted considerable overlap in the lists of iron- and Irr-regulated genes, although some genes are missing in some datasets. Among 22 operons with candidate ICE sites, 12 were differentially regulated in the Irr mutant and wild-type strains of *B. japonicum,* and 17 were either positively or negatively regulated by iron concentrations. In total, 19 operons with candidate ICE sequences were differentially expressed in the above two microarray studies.

**Table 1:**
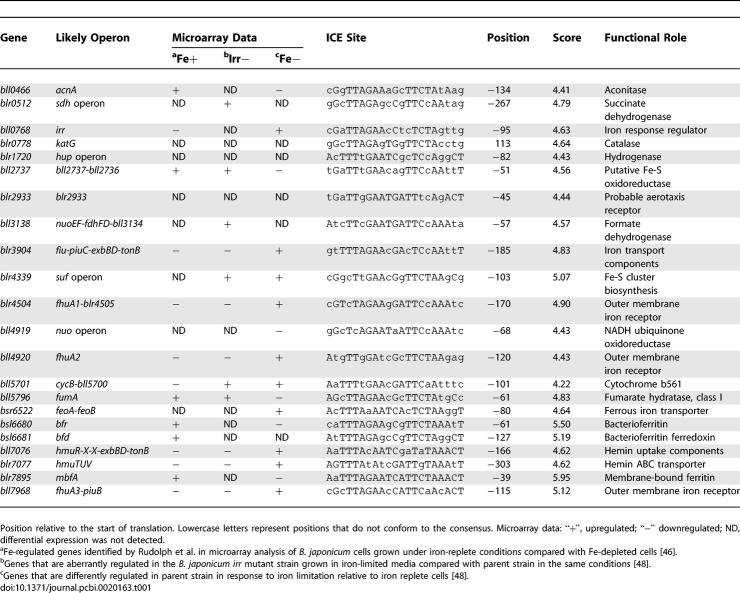
Comparison of the Predicted Irr Regulon and ICE Motifs with the Published Expression Microarray Data for Iron- and Irr-Affected Genes in Bradyrhizobium japonicum

#### Magnetospirillum magneticum.

The global gene expression analysis in the magnetotactic bacterium M. magneticum [63] revealed many iron-inducible genes, including multiple homologs of genes encoding the ferrous iron transporter FeoAB and the high-affinity iron transporter FTR1/ChpA. Based on the distribution of candidate Fur^α^-boxes in the genome of *M. magneticum,* we tentatively predict that the iron-mediated regulation of the above putative iron uptake genes is mediated by Fur^α^ (Table S6).

### Overlap between Irr and RirA Regulons

It had been shown that some genes in R. leguminosarum (for example, *suf, rirA,* and *irp6*) are under the dual control of both Irr and RirA [49]. Consistent with this, all these operons are preceded by candidate binding sites of both iron regulators (i.e., ICE- and RirA-boxes) (Tables S1 and S3). In the genomes of other α-proteobacteria that have both RirA and Irr regulons, we identified 21 other operons that were also preceded by both candidate ICE- and RirA-box motifs. These include eight operons in *A. tumefaciens (bfd-bfr, fat, fbp, fhu, fssA, irp6, rirA,* and *suf),* five in *S. meliloti (bfd-bfr, fbp, irp6, rirA,* and *suf),* and one to three operons in the *Brucella* and *Mesorhizobium* species. Unlike the RirA repressor, the Irr regulator can mediate either negative or positive control of individual target genes, depending on the respective location of its binding site and the promoter site [46,48]. In fifteen cases (mostly *suf* and *rirA* genes), the RirA-box was upstream of the ICE, suggesting a predominantly negative regulation by Irr. In the remaining nine cases, the ICE site was found upstream of the RirA-box, and thus could be potentially involved in positive gene regulation by Irr. Thus, the RirA and Irr regulons demonstrate a significant overlap in rhizobia.

### Possible Evolutionary Scenarios for the Regulatory Networks

Tentative reconstruction of the regulatory networks for the iron and manganese homeostasis genes allowed us to speculate on a most parsimonious evolutionary scenario for the Fe and Mn regulons in α-proteobacteria. Since Fur is a major global iron regulator in other subdivisions of proteobacteria (γ, β, δ, ɛ), we propose that the last common ancestor of α-proteobacteria also used a Fur-like protein to control iron metabolism. Comparative analysis of regulatory sites in this study suggests that the Fur regulon remained intact in many lineages of α-proteobacteria (we call the repressor Fur^α^), but with the notable exception of two lineages, the Rhizobiales and the Rhodobacteraceae, where Fur evolved to become a regulator of the manganese uptake in response to Mn^2+^ concentrations (and thus was called Mur). Interestingly, the consensus sequences of candidate DNA-binding sites of the Fur^α^ and Mur in α-proteobacteria still resemble each other, and show a similarity to the classical Fur-box consensus sequences from γ-proteobacteria and bacilli (Figure 4). Consistent with this, the Fur/Mur of both B. japonicum and of R. leguminosarum have been shown to recognize the classical Fur-boxes that are provided artificially, and both cloned proteins complement the E. coli Fur mutant mediating iron-dependent control of gene expression [28,32,43].

Finally, in Mesorhizobium sp. and *R. capsulatus,* the change in regulation of manganese transporters from Mur to the classical MntR was possibly achieved later by horizontal gene transfer events. A good example of a transition state of the Mn regulons is Bradyrhizobium sp. BTAi1, which has both Mur and MntR and also two paralogs of a candidate Mn uptake gene (*mntH1,* predicted to be regulated by Mur and *mntH2,* regulated by MntR).

Another possibly transitional situation is represented by the Fur-like protein of B. japonicum, which was first identified as an iron-responsive regulator of the *irr* gene [43], but, in this genomic study, was predicted to regulate both *irr* and *mntH* genes. These observations lead to the question about the natural role of Fur in B. japonicum: whether (i) the *mntH* transporter is regulated by iron; or (ii) the *irr* gene is regulated by manganese; or (iii) Fur mediates a dual response to both metals in vivo. Direct experimental work will reveal which, if any, of these predictions is correct.

In the Rhizobiales/Rhodobacteriaceae, the role of Fur in regulating iron metabolism is undertaken by two iron-responsive transcription factors, RirA and Irr, which act by sensing the physiological consequences of the metal availability, rather than its concentration per se. As suggested by Todd et al. [49], this may allow these bacteria to match their profile of gene expression in a more subtle and physiologically relevant manner than those bacteria that use Fur as their global Fe-responsive regulator. Thus, e.g., in *E. coli,* iron is sensed more directly by an interaction between Fe and Fur, but the bacteria that use RirA in combination with Irr can integrate both the availability and the biological need for the metal, as reflected by the intracellular concentrations of Fe-S and heme, two important and central Fe-containing molecules.

Among the Rhizobiales order, the RirA regulators (as well as candidate RirA-boxes) are well conserved in the Rhizobiaceae, Mesorhizobiaceae, Brucellaceae, and Bartonellaceae groups but are not present in the Bradyrhizobiaceae group. The closest relatives of these RirA proteins are the nitrite-responsive repressors NsrR from β-proteobacteria and γ-proteobacteria, and some α-proteobacteria, including Rhodobacteraceae, where they are scattered among the species and do not correlate with the presence of candidate iron regulatory motifs (Figures 1 and 3A). The involvement, if any, of these RirA homologs in the regulation of iron metabolism is unknown. We noted close positional linkage of some of these *rirA-*like hypothetical regulatory genes with the nitrosative stress response genes, suggesting that these RirA homologs may have a role analogous to NsrR for regulation of the nitrogen oxide metabolism [40]. In a most parsimonious scenario, the RirA branch was duplicated from one of these Fe-S–containing regulators of the nitrosative stress metabolism in the last common ancestor of the Rhizobiaceae after the branching of the Bradyrhizobiaceae, and evolved to sense iron concentrations, most likely via the status of the bound Fe-S cluster. In the Bradyrhizobiaceae group, Irr seems to be the only global iron regulator, and thus the Irr regulon includes most of the genes that are regulated by Fur or RirA in other lineages. An alternative scenario involves secondary loss of RirA in the Bradyrhizobiaceae with subsequent extension of the Irr regulon. The choice between these two possibilities may depend on the resolution of the α-proteobacterial taxonomy, which has not been finally established yet [64].

In the Rhodobacterales, the Iron-Rhodo-box motif appeared upstream of genes encoding iron uptake and storage proteins. The iron uptake genes are regulated only via the Iron-Rhodo-boxes, whereas for the iron storage genes the division of roles between this motif and Irr depends on the particular genome. The identity of the regulator binding to this motif is discussed in the next section. From phylogenetic analyses, we suggest that Irr began to evolve in an ancestral α-proteobacterium as an unusual member of the Fur superfamily before the branching of the Rhizobiales, Rhodobacterales, Rhodospirillales, and SAR11 lineages (Figure 2B). The reconstructed Irr regulons in the Rhodobacterales, Rhodospirillales, and Pelagibacter ubique contain only one to five target genes that are involved in the iron usage (Fe-containing enzymes), the heme and Fe-S cluster biosynthesis, and the iron storage (Figure 7). This minimal Irr regulon is significantly extended in a number of the Rhizobiales species. Furthermore, in the absence of RirA, Irr became the major iron regulatory factor in the Bradyrhizobiaceae group, where it has both positive and negative regulatory activities, depending on the location of the ICE motif relative to the promoter. In other groups of the Rhizobiales, there is a division of labor, when RirA represses the iron uptake genes and Irr activates the iron storage and usage genes in Fe-replete conditions. However, this regulatory strategy is not universal, since in some species (e.g., *Agrobacterium*), the candidate Irr regulon became extended to include many RirA-controlled operons (Figure 7). Finally, the obligate intracellular pathogens from the Rickettsiales order (*Rickettsia* and *Ehrlichia*) lack the RirA, Irr, and Fur/Mur regulons, and retain only the IscR-mediated control of their Fe-S synthesis *suf* operons. A small oceanic bacterium from the Rickettsiales order, *Pelagibacter ubique,* is predicted to have the minimal iron regulatory system, which includes only the ferric iron transporter and rubrerythrin genes under possible control of Fur^α^ and Irr, respectively, and also the candidate IscR-regulated *suf* operon.

### Evolution of Motifs and the Identity of the Transcription Factor that recognizes the Rhodo-Iron-Box

The reconstructed iron regulatory network suggests that bacteria from the Rhodobacteraceae group have at least two different iron regulons, the Irr regulon controlling mostly the iron usage genes and another candidate regulon, which operates via the Iron-Rhodo-box motif and includes most of the iron uptake and storage genes (Figure 7). The Iron-Rhodo-box motif has a significant similarity to the RirA-box motif in the Rhizobiales (Figure 4A and 4B). However, the absence of close RirA homologs in twelve analysed species of the Rhodobacteraceae suggests that another transcription factor mediates the global control of iron transport genes.

One reasonable candidate for this role is the Fe-S synthesis regulator IscR, which is present in all Rhodobacteraceae species as well as in other lineages of α-proteobacteria except the Rhizobiales group (Figure 1). IscR and RirA regulators belong to the same protein superfamily (Rrf2). The predicted DNA-recognition motifs of RirA from the Rhizobiales, and IscR from the Rhodobacteraceae, are rather similar (Figure 4B and 4E), and, in some α-proteobacteria (e.g., in *Loktanella, Rhodobacter, Roseobacter,* and *Silicibacter* species), the Iron-Rhodo-box profile identifies the same candidate sites as the IscR profile. Intriguingly, the Iron-Rhodo-box profile identifies the candidate IscR site preceding the *iscR-suf* operon in Pelagibacter ubique.

The tantalizing hypothesis that IscR may have a more global role in the regulation of iron homeostasis in the Rhodobacteraceae is supported by the similarity of DNA motifs, the complementary distribution of the RirA and IscR factors in the analysed genomes, and the multiple alignment of IscR protein sequences. The IscR proteins from the Rhodobacteraceae group have an interesting feature that distinguishes them from other IscR regulators. Three Cys residues involved in Fe-S cluster binding, which are highly conserved in most other IscR proteins, are absent from the IscR proteins of the Rhodobacteraceae. Thus we tentatively propose the following possible strategy of the iron-dependent transcriptional control in the Rhodobacteraceae lineage. The IscR protein recognizes Iron-Rhodo-boxes and thus regulates the iron uptake and storage genes. Another iron-responsive transcription factor (Irr) regulates the iron usage genes, including the Fe-S synthesis *iscR-suf* operon, thus forming a possible regulatory cascade with IscR.

**Figure 9:**
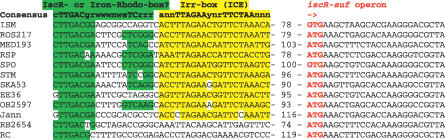
Multiple Alignment of the Upstream Regions of the *iscR-suf* Operons in the Rhodobacteraceae Species

In the above model, it is not clear that IscR regulates expression of the cognate *iscR-suf* operon. Multiple alignment of the *iscR-suf* upstream regions from 12 analyzed Rhodobacteraceae genomes (Figure 9) shows a strong conservation of the candidate Irr recognition motif (ICE) in all but two species, as well as an additional highly conserved sequence 5′-cTTGACgr-3′ (“r” denotes a purine) located 12 nt upsteam of the ICE. The latter sequence resembles the left half of the IscR motif. At the same time, only some of these sequences have the right half of the candidate IscR site, suggesting that possible autoregulation of *iscR-suf* operon by IscR is not well-conserved among the Rhodobacteraceae. Another possibility is that IscR does not regulate the *iscR-suf* operon, and the 5′-cTTGACgr-3′ sequence is conserved for some other reason, e.g., because it is the “–35”-box of a promoter (the “–35”-box consensus is TTGACA).

The sequence logos constructed for candidate Fur^α^-boxes, RirA-boxes, Iron-Rhodo-boxes, and IscR-boxes in α-proteobacteria all show a faint similarity to each other (consensus TG-N_11_-CA, see Figure 4) that is also shared by both the conventional Fur-boxes in γ-proteobacteria. Thus, very speculatively, the iron-regulatory signals in α-proteobacteria may have evolved from a canonical Fur*-*box. Indeed, theoretical calculations demonstrate that sites even weakly conforming to the requirements of the binding protein may provide sufficient initial advantage for the positive selection to come into action and perfect them to a higher-affinity state [65].

### Concluding Remarks

**Figure 10:**
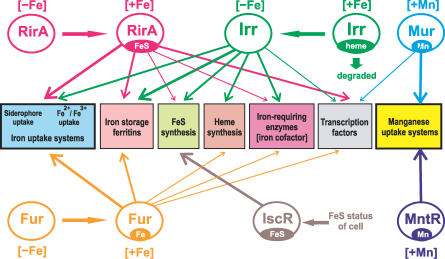
Combined Regulatory Network for Iron and Manganese Homeostasis Genes in α-Proteobacteria The connecting lines denote regulatory interactions, with the thickness reflecting the frequency of the interaction in the analyzed genomes.

The results of this comparative genomics study demonstrate significant novelty and variability of iron and manganese regulatory networks in α-proteobacteria, both in their proposed mechanisms (Figure 10) and in the functional content of target genes (Figure 7). We recognize that nearly all direct work on regulation of iron homeostasis in this group has been done only in three rhizobia species [50]. However, this information, complemented by the computational ab initio reconstruction of the regulons, allows us to form some general conclusions and several speculations about the nature of this process in the “alphas.”

Although the emerging overall picture of regulatory interactions seems to be rather consistent and robust, it is unlikely that the described regulatory network is responsible for all the Fe-responsive gene regulation in α-proteobacteria. Even in *Bradyrhizobium* and *Rhizobium,* two genera that have been studied more than the others, several genes are differentially expressed in Fe-replete and Fe-depleted cells but not via any of the regulators studied so far.

Given the explosion in bacterial genome sequencing, with more than 1,000 complete and ongoing genome projects, more and more functional analyses are being done in silico*.* While these, of course, can point the way to revealing patterns and phenomena, there is no escape from the need for direct experimentation. We believe that numerous bioinformatic predictions of components of the iron regulatory network in the α-proteobacteria presented in this work are sufficiently interesting to warrant experimental verification.

## Materials and Methods

Complete and partial bacterial genomes were downloaded from GenBank [66]. Preliminary sequence data were also obtained from the Web sites of the Institute for Genomic Research (http://www.tigr.org), the Wellcome Trust Sanger Institute (http://www.sanger.ac.uk), and the US Department of Energy Joint Genome Institute (http://jgi.doe.gov). The gene identifiers from Genbank are used throughout. Genome abbreviations are listed in Figure 1. Protein similarity searches were done using the Smith-Waterman algorithm implemented in the GenomeExplorer program [67]. Orthologous proteins, or homologs that diverged following a speciation event, were defined by the bidirectional best hits criterion [68] and named by either the common name of a characterized protein or by an identifier in the Clusters of Orthologous Groups (COG) database for uncharacterized proteins [69]. The phylogenetic trees were constructed by the maximum likelihood method implemented in the PROML program of the PHYLIP package [70] using multiple sequence alignments of protein sequences produced by the CLUSTALX program [71]. In addition we used Psi-BLAST [72] (http://www.ncbi.nlm.nih.gov/BLAST) to conduct long-range similarity searches, the PFAM [73] (http://www.sanger.ac.uk/Software/Pfam/) and Conserved Domain databases [74] (http://www.ncbi.nlm.nih.gov/Structure/cdd/cdd.shtml) to identify conserved functional domains, and TMpred [75] (http://www.ch.embnet.org/software/TMPRED_form.html) to predict transmembrane segments. The conserved positional clusters of genes were analyzed by the SEED comparative genomic database (http://theseed.uchicago.edu/FIG/index.cgi) [76].

To identify candidate regulatory motifs, we started from sets of potentially co-regulated genes (using previous experimental and general functional considerations). An iterative motif detection procedure implemented in the program SignalX was used to identify common regulatory DNA motifs in a set of upstream gene fragments and to construct the motif recognition profiles as previously described [77]*.* For the RirA regulon, we started from a training set of the upstream regions of known RirA targets in R. leguminosarum and their orthologs in other RirA-encoding genomes [36,41]. For the candidate iron regulon in the Rhodobacteraceae, we used a set of upstream regions of the iron uptake and storage genes from 12 available genomes to construct the conserved recognition profile (Figure 4). For the Irr regulon, we started from a training set of the upstream regions of iron uptake and storage genes in the Bradyrhizobiaceae, and from the training set of known Irr/iron-regulated genes in R. leguminosarum [49] and their orthologs in other Irr-encoding genomes from the Rhizobiaceae and Rhodobacteraceae groups. Finally, for each representative of α-proteobacteria outside of the Rhizobiales and Rhodobacteraceae groups, we used a separate training set of the upstream regions of iron uptake and storage genes to construct the Fur^α^-box profile. The constructed group- or species-specific recognition rules were used to scan a subset of genomes of the α-proteobacteria that contains the respective regulator. Positional nucleotide weights in the recognition profile and z*-*scores of candidate sites were calculated as the sum of the respective positional nucleotide weights (as previously described in [78]). Genome scanning for specific regulatory motifs by the GenomeExplorer software [67] produced gene sets with candidate regulatory sites in the upstream regions (Tables S1–S6). The threshold for the site search was defined as the lowest score observed in the training set (indicated in red numbers for each analyzed genome in the “Score” column of Tables S1–S6). The threshold choice was adequate in our cases, since very few clear false positives were encountered, and, on the other hand, most functionally relevant genes were found to belong to at least one of the studied iron regulons. The upstream regions of genes that are orthologous to genes containing regulatory sites of any of the studied iron-responsive regulators were examined for candidate sites even if these were not detected automatically with a given threshold (weak regulatory sites with scores below the threshold are underlined in Tables S1–S6). Sequence logos for the derived group- or species-specific regulatory motifs were drawn using the WebLogo package version 2.6 [79] (http://weblogo.berkeley.edu).

## Supporting Information

Figure S1 Species-Specific Deviations in Fur^α^ Recognition Signals of α-Proteobacteria (163 KB PDF)

Figure S2 Genome Context of the Heme Uptake and Utilization Genes in α*-*Proteobacteria (44 KB PDF)

Table S1 Candidate RirA-Box Iron Regulons in Rhizobiaceae, Mesorhizobiaceae, *Brucella,* and *Bartonella* Species (18 KB PDF)

Table S2 Candidate Iron-Rhodo-Box Regulons in the Rhodobacteraceae (18 KB PDF)

Table S3 Candidate Irr-Box (ICE) Regulons in α-Proteobacteria (26 KB PDF)

Table S4 Candidate Mur-Box (MRS) Regulons in α-Proteobacteria (10 KB PDF)

Table S5 Candidate MntR-Box Regulons in α-Proteobacteria (5 KB PDF)

Table S6 Candidate Fur^α^-Box Regulons in α-Proteobacteria (19 KB PDF)

Table S7 Candidate IscR-Box Regulons in α-Proteobacteria (77 KB PDF)

## Abbreviations

ABC: ATP-binding cassette
Fur: ferric uptake regulator
ICE: iron control element
IRO: iron-responsive operators
MRS: Mur-responsive sequence
Mur: manganese uptake regulator
OMP: outer membrane iron receptors
