# Supplementary material for: Computational Reconstruction of Iron- and Manganese-Responsive Transcriptional Networks in α-Proteobacteria
Source: PLoS Comput Biol. 2006 Dec 15;2(12):e163. doi: 10.1371/journal.pcbi.0020163 (PMC1698941; doi:10.1371/journal.pcbi.0020163)
Supplement: Supplementary file 2 [file pcbi.0020163.sg002.pdf]

Supplementary Figure S2.

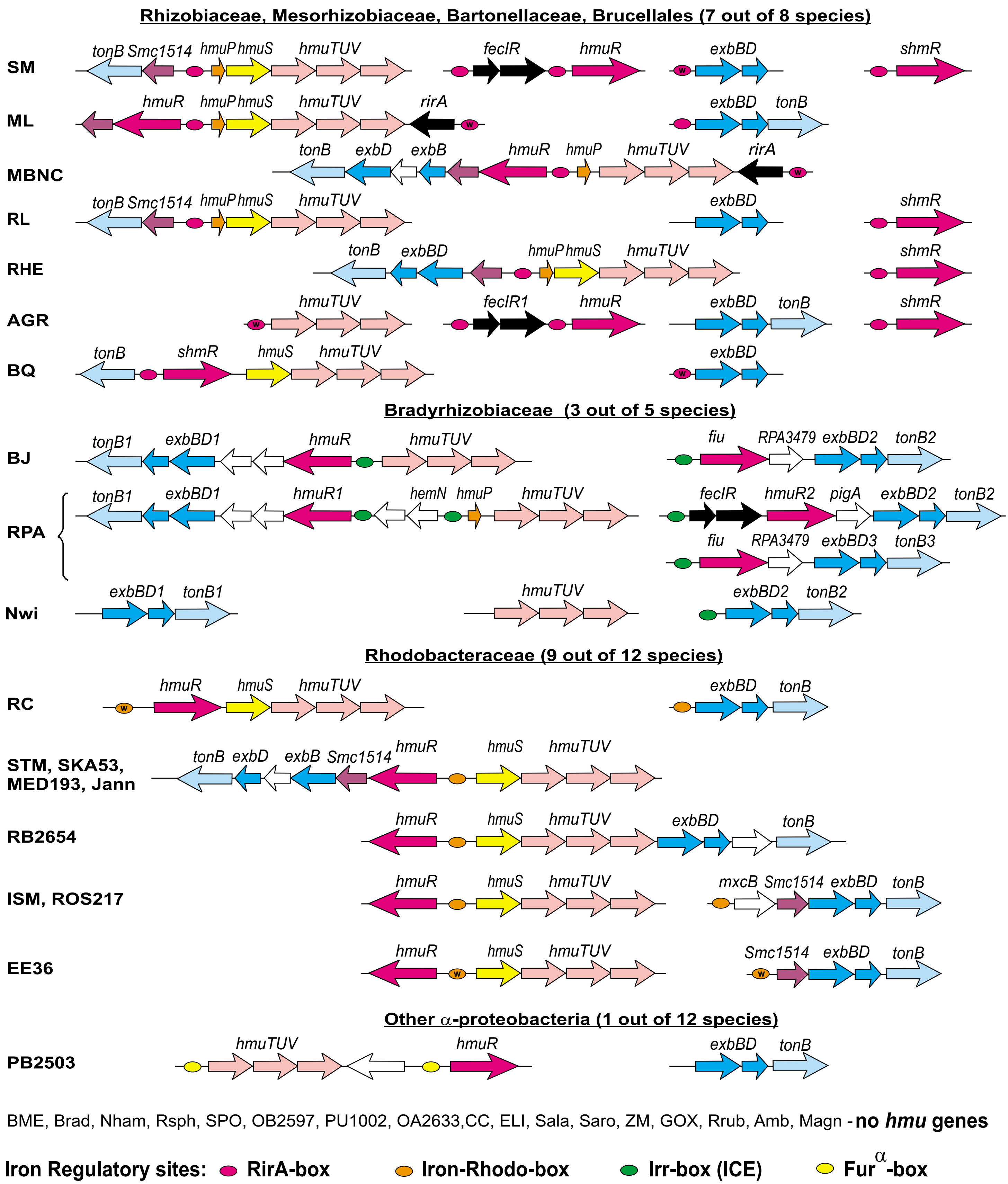

Weak sites with score below the threshold are marked by 'w'.
Homologous genes and groups of genes are marked by matching colors.
Genome abbreviations are described in Figure 1.
