# Supplementary material for: Computational Reconstruction of Iron- and Manganese-Responsive Transcriptional Networks in α-Proteobacteria
Source: PLoS Comput Biol. 2006 Dec 15;2(12):e163. doi: 10.1371/journal.pcbi.0020163 (PMC1698941; doi:10.1371/journal.pcbi.0020163)
Supplement: Supplementary file 3 [file pcbi.0020163.st001.pdf]

Suppl. Table S1. The RirA regulon in alpha-proteobacteria

Locus ID	Operon	Position	Score	RirA-box	Function
Sinerhizobium meliloti					
SMc01657	*fhuA2-fhuF-irp6A2*	-94	5.34	CATGACTtaAAaAGTCATG	ferrichrome iron receptor, ferric iron reductase, Ferrisiderophore-binding protein
		-51	4.01	CATGAgTgaTtagcTCAcc	
SMa2412	*rhrA*	-107	4.88	CAaGACTATTtaAGTCATG	transcriptional activator of rhizobactin biosynthesis and transport
SMa2414	*rhtA-iucB(vbsA)*	-80	4.88	CATGACTtaAATAGTCtTG	rhizobactin receptor, siderophore biosynthesis
SMc01747	*hmuPSTUV*	-134	4.82	tATGAgTAaTgaAGTCAaG	hemin ABC transporter
SMc01514	*SMc01514-tonB*	-114	4.82	CtTGACTtcAtTAcTCATa	hypothetical protein, iron transport component TonB
SMc02889	*rhtX2 - viuA*	-65	4.77	CcTGAaTgcAATAGTCAaG	ferrisiderophore permease and receptor
SMc00529	*sufS2-sufBCDS1XA*	-164	4.74	CtTGACTAaAccAGTCAgc	Fe-S cluster assembly
SMc04203	*fecI-fecR*	-171	4.68	CATGAtTATctcAaTCAac	iron transport sigma factor and sensor
SMb21432	*irp6A1-irp6BC*	-58	4.68	gtTGACcggTAaAGTCATG	ferrisiderophore ABC transporter
SMc00045	*cycF-cycG*	-183	4.57	gATGAgTtcTATAtTCATa	cytochrome c-556, diheme cytochrome c-type [heme]
SMa2337	*rhtX-rhbABCDEF*	-60	4.47	gATGACggcAAcAcTCATG	rhizobactin siderophore transporter, and biosynthesis genes
SMc00592		-54	4.37	CtTGACgATcAaAGTCAgc	similar to AMP-dependent synthetase / ligase:Acyltransferase 3
SMc02726	*shmR*	-140	4.26	atTGACaATcAcAcTCATG	hemin receptor
		-53	3.84	CtTGAgTtcgccgcTCAac	
SMc02727		-89	3.84	gtTGAgcggcgaAcTCAaG	hypothetical protein
		-2	4.26	CATGAgTgTgATtGTCAat	
SMc04205	*hmuR*	-120	4.26	tATGACatccgTttTCAaG	heme receptor
SMc02085	*exbB-exbD*	-182	4.20	CtTGAtTATTgTAcTCcac	components for TonB-dependent iron transporters
SMc01611	*fhuA1*	-86	4.08	gtTGAtctagAcAcTCATc	ferrichrome iron receptor
		-62	4.19	gtTGACagTAccgGTCAcG	
SMa1746	*fepBDGC-mxcB*	-178	4.09	CATGAaagcTtcgcTCAac	ferrienterobactin ABC transporter, siderophore-interacting protein
SMa1747	*fhuA3*	-103	4.09	gtTGAgcgaAgcttTCATG	ferrichrome iron receptor
SMa1749	*araX1*	-115	4.01	aATGAgaATAcgAGTCAac	siderophore uptake AraC-type regulator
SMc00784	*fbpA*	-46	3.97	tATGAtTgactaAGTtAaG	ABC-type Fe3+ transport system, periplasmic component
SMc00785	*rirA*	-172	3.97	CtTaACTtagtcAaTCATa	iron-responsive regulator RirA
SMc03787	*bfd-bfr*	-67	3.79	gtTGACggcAAatGTCcTc	bacterioferritin ferredoxin, bacterioferritin
Expression data:	***RirA-repressed genes (Chao et al., 2006)***				
Mesorhizobium loti					
msl1154	*hmuPSTUV*	-82	5.06	CATGAtTATTcaAGTCAaG	hemin ABC transporter
mlr1155	*hmuR-SMc01514*	-84	5.06	CtTGACTtgAATAaTCATG	heme receptor, hypothetical protein
mll4168	*exbB-exbD-tonB*	-83	4.99	gtTGACTgTTgcAGTCATc	components for TonB-dependent iron transporters
		-58	4.89	gtTGACTtgTATAcTCATG	
mlr3495	*fbpABC*	-68	4.80	gtTGACTATTcgtGTCATG	ferric cations ABC transporter
mll6832		-260	4.74	CtTGAaTAaAtctaTCAaG	hypothetical protein
mll5668		-57	4.72	CtTGACTAaAAaAcTCAcc	transcriptional regulator, ArsR family
mll0429		-318	4.60	CATGAtTcaAAaAcTCAac	hypothetical protein
mlr1147	*rirA*	-173	4.46	gtTGACgAgAATAtTCATc	iron-responsive regulator RirA
mlr5523	*irpA- bfd-bfr*	-257	3.89	gtTGACTtcTgTAGTtaG	iron-regulated protein A, bacterioferritin ferredoxin, bacterioferritin

***Mesorhizobium* sp. BNC1**					
MBNC03002200	*hmuPTUV*	-84	4.86	gtTGAtTATTgatGTCAaG	hemin ABC transport system
MBNC03002201	*hmuR-SMc01514-exbBD-tonB*	-172	4.86	CtTGACatcAATAaTCAac	Fe receptor, conserved protein, TonB-dependent transporter components
MBNC03003078	*bfd-bfr*	-183	4.79	CATGACctTgAaAGTCAaG	bacterioferritin ferredoxin, bacterioferritin
MBNC03004224	*fhuA-rhtX-mxcB*	-148	4.78	gtTGACatTgATAaTCATG	ferrichrome iron receptor, MFS permease, siderophore-interacting protein
MBNC03004482	*chpA -X- ftr -COG0348*	-183	4.70	gtTGACctTTcaAGTCATG	involved in high-affinity Fe transport, High-affinity Fe permease, Polyferredoxin
MBNC03003400	*fbpAB*	-154	4.19	CtTGACTAcTATAGTttaG	ferric cations ABC transporter
MBNC03003363	*fecB*	-75	4.09	ggTGAtgtTgAcAGTCAaG	iron(III) dicitrate-binding protein
		-29	4.06	tcTGACcATgcTtGTCAaG	
MBNC03003364	*fecCDE*	-91	4.06	CtTGACaAgcATgGTCAga	iron(III) dicitrate ABC transporter, permease subunits, ATPase
		-45	4.09	CtTGACTgTcAacaTCAcc	
MBNC03002196	*rirA*	-136	3.60	CtgGACctaTAgcGTCATc	iron-responsive regulator RirA
Rhizobium leguminosarum					
pRL120322	*fhuA1-fhuF*	-19	5.10	gATGACTAaAATAaTCATc	ferrichrome iron receptor, ferric iron reductase
RL4584	*fbpABC*	-25	4.75	tATGAaataTtTAGTCAaG	ferric cations ABC transporter
pRL100314		6	4.72	CATGACaAgAgcAcTCAaG	hypothetical protein
RL3522	*shmR*	-178	4.71	CATGAtagaTATAtTCAac	hemin receptor
		-142	4.60	gtTGACaATgcTAcTCATG	
RL3695	*SMc01514-tonB*	-114	4.71	CtTGACTtTctTAcTCATa	conserved protein, iron transport component TonB
RL3696	*hmuPSTUV*	-81	4.71	tATGAgTAagAaAGTCAaG	hemin ABC transporter
RL2713	*irp6ABC*	-66	4.68	CtTGAtTcaTtaAGTCATa	ferrisiderophore ABC transporter
RL2583	*sufS2-sufBCDS1XA*	-164	4.63	CtTGACTAgTgTtGTCAgc	Fe-S cluster assembly
pRL120319	*rpoI*	-134	4.28	CtTGACTccTgTtaTCAcc	iron transport sigma factor
pRL120320	*vbsC*	-48	4.28	ggTGAtaAcAggAGTCAaG	vicibactin biosynthesis
pRL100328	*fhuCDB*	-63	4.09	gtTGAtTgccgctcTCATc	ferrichrome iron transporter
pRL120713	*btuFCD*	-290	4.01	gtTGAtTtgTtTgGTCtTG	siderophore ABC transporter (SBP, permease) similar to B12 ABC transporter
pRL100325	*fhuA2*	-96	4.00	gtTGACTccAATtcTCcTG	ferrichrome iron receptor
001_4255	*fumC*	-120	4.00	CATGACTtccgattTCAat	fumarate hydratase C
RL0777	*rirA*	-86	3.80	gcTGAaTgcgAattTCATa	iron-responsive regulator RirA
pRL120315	*vbsGSO*	-88	3.98	gAaGAaaAaAATtcTCATc	vicibactin biosynthesis
pRL120316	*vbsADL*	-124	3.98	gATGAgaATTtTttTCtTc	vicibactin biosynthesis
RL4696-RL4695	*bfd-bfr*	-128	3.59	CtTGACagccAccaTCtTc	bacterioferritin ferredoxin, bacterioferritin
		-97	3.56	CAcGAtagTcAgtGTCATG	
Experimental data:	***Fe-RirA-repressed operons (Yeoman et al., 2004;Todd et al., 2002; 2006)***				
Rhizobium etli					
RHE_CH03076	*shmR*	-142	4.96	gtTGACaAcAgaAGTCATG	hemin receptor
RHE_CH03266	*Smc01514-exbBD-tonB*	-117	4.71	CtTGACTtTctTAcTCATa	conserved protein, components of TonB-dependent transporters
RHE_CH03267	*hmuPSTUV*	-79	4.71	tATGAgTAagAaAGTCAaG	hemin ABC transporter
RHE_CH04056	*fbpABC*	-69	4.66	CtTGAaataggTAGTCAaG	ferric cations ABC transporter
RHE_CH02254	*sufS2-sufBCDS1XA*	-237	4.56	CtTGACTAacgTtGTCAgc	Fe-S cluster assembly
RHE_CH02383	*fumC*	-120	4.11	CATGACTgTgATcaTCAat	fumarate hydratase C
RHE_CH00735	*rirA*	-88	3.8	gcTGAaTgcgAattTCATa	iron-responsive regulator RirA

Agrobacterium tumefaciens					
AGR_L_2869	*fecIR1*	-132	5.04	CtTGAtaATAATtGTCATG	iron transport sigma factor and sensor
AGR_C_616	*fssA*	-108	4.87	CtTGAaTgTAATtaTCATG	hypothetical Fe-S scaffold protein
AGR_pAT_635	*mocC*	-2	4.80	CATGAaTATTAcAGTCAcc	myo-inositol 2-dehydrogenase
AGR_C_720	*araX*	-42	4.75	CATGAaTAgAtTtaTCATG	siderophore uptake AraC-type regulator
AGR_L_309	*exsA*	-217	4.73	CATGACatcAtcAtTCATc	ATP-binding transport protein (EPS I biosynthesis)
AGR_C_1984		0	4.72	CATGACTATcgatcTCAaG	short chain dehydrogenase
AGR_L_2862	*irp6AABC*	-63	4.68	CtTGACTAgTAcAGTCAga	ferrisiderophore ABC transporter
AGR_C_4156	*shmR*	-105	4.76	CATGAtTcaAAaAGTCATa	hemin receptor
		-78	4.52	gtTGACagTgATcGTCATG	
AGR_C_344	*rirA*	-145	4.62	CtTGACTtgTtTgtTCATG	iron-responsive regulator RirA
AGR_C_346	*fbpA2*	-2	4.62	CATGAacAaAcaAGTCAaG	ferric cations ABC transporter
AGR_L_2323	*AGR_L_2323-2304-viuA-fecBCI*	-173	4.54	CtTGAaTcgctTAGTCAaG	siderophore biosynthesis, iron (III) dicitrate ABC transporter
AGR_L_2326	*AGR_L_2326-2334*	-107	4.54	CtTGACTAagcgAtTCAaG	siderophore biosynthesis cluster
AGR_L_2875	*hmuR*	-270	4.44	gtTGAgTAaTccgGTCAaG	heme receptor
AGR_C_5028	*bfd-bfr*	-70	4.39	CtTGACatTTActGTCcTG	bacterioferritin ferredoxin, bacterioferritin
AGR_C_3350	*sufS2-sufBCDS1XA*	0	4.26	CtTGACgAactgtGTCATc	Fe-S cluster assembly
AGR_L_1661	*fhuADBC*	-61	4.24	gATGACTtaAATtcTCcaG	ferrichrome iron receptor, ABC transporter
AGR_C_715	*fbpAB*	-28	4.21	CtTGAtTtgcgccGTCATG	ferric cations ABC transporter
AGR_C_718	*fhuA2*	-29	4.05	CcTGAtgtaAAcAcTCATc	ferrichrome iron receptor
AGR_C_4467	*hmuTUV*	-22	3.58	CATGtgcAaAtaAcTCAaG	hemin ABC transporter
AGR_pAT_442	*fecIR2- fhuA3-fepB-mxcB-fepDC*	-396	4.00	gcTGAaTAcAgctaTCAaa	ferrienterobactin ABC transporter, siderophore-interacting protein
		-87	3.96	CATGAgTtTTcTtcTCtac	
AGR_L_2290	*fecIR3*	-218	4.26	CATGAtaAaAATtGTCcTc	
AGR_C_4488	*fatBDCE*	10	3.83	gcTGACagcgAacaTCAaG	ferric anguibactin ABC transporter
Brucella melitensis					
BMEI0542		-132	4.92	CATGAtTAacATAaTCATG	hypothetical protein
BMEI0543		-162	4.92	CATGAtTATgtTAaTCATG	choloylglycine hydrolase
BMEII0105	*OMP*	-262	4.85	tATGAaTgTAtTAGTCATc	outer membrane iron receptor
BMEII1051	*fumC*	-80	4.53	CtTGACatTTATAtTCAca	fumarate hydratase C
BMEI0365	*exbB-exbD-tonB*	-163	4.46	CATGACTtgAAgtaTCATa	components for TonB-dependent iron transporters
BMEII0077	*dhbCEBAD*	-55	4.38	CgTGACTggTtcttTCAaG	2,3-dihydroxybenzoic acid biosynthesis (brucebactin synthesis)
BMEII0584	*fbpAB*	-53	4.15	CcTGAtccaTATAcTCATG	ferric cations ABC transporter
BMEI1042	*sufBCDS1X*	-160	4.03	CtTGAaTttTaTtGgCAaa	Fe-S cluster assembly
BMEII0705	*bfd-bfr*	-344	4.00	CATGgaTATTtaAcTCATa	bacterioferritin ferredoxin, bacterioferritin
BMEII0707	*rirA*	-182	3.72	ggTGAgaAaAggttTCATa	iron-responsive regulator RirA
BMEII0607	*fatBDCE*	-88	3.49	gtTGACTATAAagcaCAgG	ferric anguibactin ABC transporter
Experimental data: ***Iron-responsive regulation (Bellaire et al., 2003)***					
Bartonella quintana					
BQ04160	*shmR-hmuSTUV*	-157	4.93	CtTGACTgcTtTtaTCATG	hemin receptor and ABC transporter
BQ04170	*tonB*	-101	4.93	CATGAtaAaAgcAGTCAaG	components for TonB-dependent iron transporters
BQ02630		-53	4.65	ttTGAaTtTTtaAtTCATG	Phage related lysozyme
BQ05940	*sufS2-sufBCDS1XA*	-76	4.40	ttTGAgTgcAtTttTCAaG	Fe-S cluster assembly

BQ02420	*hbpAB*	-261	4.26	CAgGACTcaTATtaTCATG	hemin binding proteins
BQ11710	*exbB-exbD*	-342	4.05	ttTGAtaAaAtcAaTCtTG	components for TonB-dependent iron transporters
BQ10310	*fatDCE*	-75	3.74	CATGAaataAAgttTtAaG	ferric anguibactin ABC transporter
BQ10300	*fatBDCE*	-35	3.44	gATGAtcAaAtatcTgAaa	ferric anguibactin ABC transporter

Color code for functional roles of genes:

Genes involved in iron uptake

Genes involved in iron storage

Genes involved in iron utilization pathways (synthesis of Fe-S, heme; iron-containing enzymes)

Transcription factors (rirA, araX, fecIR, irr)

Genes with other or uncertain functional roles
