# Supplementary material for: Computational Reconstruction of Iron- and Manganese-Responsive Transcriptional Networks in α-Proteobacteria
Source: PLoS Comput Biol. 2006 Dec 15;2(12):e163. doi: 10.1371/journal.pcbi.0020163 (PMC1698941; doi:10.1371/journal.pcbi.0020163)
Supplement: Supplementary file 4 [file pcbi.0020163.st002.pdf]

Suppl. Table S2. The iron regulon in Rhodobacteraceae

Locus ID	Operon	Position	Score	Iron-Rhodo-box	Function
Rhodobacter sphaeroides					
RSP_6006	*hmuP-irpA -bfd-bfr*	-199	4.53	TCcGACTgATTcAGTCAcc	hemin uptake protein, iron-regulated protein A, ferredoxin, bacterioferritin
RSP_3416	*fecBCDE*	-156	4.43	TCcGACTgtTcTtGTCgGA	iron(III) dicitrate ABC transporter
RSP_3417	*OMP1*	-289	4.43	TCcGACaAgAacAGTCgGA	TonB-dependent siderophore receptor
RSP_2913	*fbpA*	-81	4.38	gtTGACTgATagAcTCAGc	ferric cations ABC transporter
RSP_1545	*RSP_1545-1544-1543*	-64	4.33	TCcGAtTgAAagAcTCgGg	thiol oxidoreductase, periplasmic protein, hypothetical protein
RSP_0443	*iscR -sufSBCD*	-120	4.22	ctTGACaAAAacgcTCgGA	Fe-S cluster assembly
Rsph03003247	*fecI-fecR*	-69	4.16	gCcGAgcgATaTtGTCgGg	iron transport sigma factor and sensor
RSP_0920	*exbB-exbD-tonB*	-133	3.97	ctTGACTgtTTTcGTaAac	components for TonB-dependent iron transporters
Rhodobacter capsulatus					
RRC03231	*hemN2*	-143	4.78	ctTGACaAAATTAcTCgGA	paralog of oxygen-independent coproporphyrinogen III oxidase
RRC03288	*X-feoAB*	-66	4.22	cCatACTAtTTcAGTCAGg	ferrous iron transporter
		-35	4.5	ctTGACTtATccAGTCAGA	
RRC04402	*viuA*	-242	4.42	gCcGAtaAAAaTAcTCgGA	vibriobactin receptor precursor
RRC04403	*fepBDGC*	-74	4.42	TCcGAgTAtTTTtaTCgGc	ferrienterobactin ABC transporter
RRC04414	*araX2*	-421	4.41	TgTGACaAAAaTAGTCAac	AraC-type regulator for siderophore uptake (next to fhuA3<>fepBDGC2-mxcB)
RRC04415	*araX3*	-50	4.41	gtTGACTAtTTTtGTCAcA	AraC-type regulator for siderophore uptake
RRC02269	*araX4 - fhuA1-fhuDBC*	-56	4.38	gtTGAtTgtTTctGTCAGA	ferrichrome iron receptor, ABC transporter
RRC00211	*FTR1-COG2837-chpA*	-32	4.28	TCcGACaAAATaAaTaAGg	High-affinity Fe2+ transport component and permease, peroxidase
RRC04484	*araX1 -irp6ABC*	-100	4.26	CtTGtgTaATTttGTCAGG	ferrisiderophore ABC transporter
RRC04485	*fhuA2-fes*	-40	4.26	cCTGACaAAATTAcaCAag	ferrichrome iron receptor, enterochelin esterase
RRC01804	*X- hmuP*	-85	4.26	cCcGACTAAAagAtTCgGg	hemin uptake protein
RRC01044	*fbpAB*	-83	4.2	ctTGACggATTTAGTCAtg	ferric cations ABC transporter
RRC02196	*troA-fecD*	-34	4.18	atcGACTctTTcAcTCgGA	iron siderophore ABC transporter
RRC04521	*hmuRSTUV*	-98	4.17	ctTGACaAAATgAcTagGA	hemin receptor, ABC transporter
RRC04412	*fepBCDE2*	-121	4.11	aCTGAtggtTTTtGTCAGA	ferrienterobactin ABC transporter
RRC04413	*fhuA3*	-173	4.11	TCTGACaAAAaccaTCAGt	ferrichrome iron receptor
RRC01434	*exbB-exbD-tonB*	-92	4.11	ctTGACaAgTTcAcTCAac	components for TonB-dependent iron transporters
RRC00380	*fssA*	-55	3.93	ctTGACggtTTcgGTCgGc	hypothetical Fe-S scaffold protein
***Silicibacter s* p. TM1040**					
TM1040_0124	*fbpA*	-125	4.77	TCTGACTgAATTtaTCgGA	ferric cations ABC transporter
TM1040_2198	*hmuP-irpA*	-259	4.56	TCTGACcAAATaAaTCAGg	hemin uptake protein, iron-regulated protein A
TM1040_2633		-117	4.38	TCcGACaAAAaTAcTagGg	hypothetical protein A, conserved regulation in MED193 and ISM
TM1040_3525	*sdhCDAB*	-174	4.08	TCTGACTgtTaTAcTtgGg	succinate dehydrogenase
TM1040_2200	*bfd-bfr*	-72	4.08	gCTtAtTctTTTAcTCgGA	bacterioferritin ferredoxin, bacterioferritin
TM1040_0347	*hmuR-Smc01514-exbBD-tonB*	-190	3.91	ctTGACagATTTAaTaAat	hemin receptor, conserved protein, components of TonB-dependent transporters
TM1040_0346	*hmuSTUV*	-127	3.91	atTtAtTAAATctGTCAag	hemin ABC transporter
TM1040_1240	*iscR -sufSBCD*	-138	3.7	ctTGACgAATTTtaTCcGg	Fe-S cluster assembly
Silicibacter pomeroyi					
SPO0086	*irpA*	-89	4.94	aCTGACaAATTTAGTCAGA	iron-regulated protein A

SPO3139		-32	4.68	TCcGACTttAacAGTCgGA	similar to truncated hemoglobins
SPO3287	*fbpA*	-51	4.56	cCcGACTgAAaTAaTCAGt	ferric cations ABC transporter
		-2	4.67	atTGACTAtTTcAGTCgGA	
SPO0789	*hmuP-hmuX**	-100	4.44	ggcGACaAAAaTAGTCAGg	hemin uptake protein, hypothetical conserved protein
SPO2025	*iscR -sufSBCD*	-140	4.04	ctTGACgAATTcccTCgGg	Fe-S cluster assembly
SPO3288	*fbpB*	-26	4.06	TCcGACTAAAcTgGTCgat	ferric cations ABC transporter
***Jannaschia* sp. CC51**					
Jann_0118	*fbpA*	-38	5.05	cCTGACTgtTTTAGTCgGA	ferric cations ABC transporter
Jann_3275	*hmuP-Jann3275*	-82	4.95	tCTGAgTAAAaTAcTCAGG	COG4256: hemin uptake protein, hypothetical Fe-S protein
		-54	4.24	gCatACTAtTTTAGTCAGG	
Jann_2122	*fecB*	-60	4.15	TCTaACTAtTTctGTCAGc	iron(III) dicitrate-binding protein
		-35	4.86	TCTGACTAAAaTAcTCAac	
Jann_2123	*fecCDE*	-60	4.86	gtTGAgTAtTTTAGTCAGA	iron(III) dicitrate ABC transporter, permease subunits, ATPase
		-35	4.15	gCTGACagAAaTAGTtAGA	
Jann_3277	Jann_3277-*dps*	-30	4.66	cCTGAgTAATacAcTCAag	hypothetical protein, DNA-binding ferritin-like protein Dps
Jann_1368	*irpA*	-254	4.58	TCTGACaAAAcaAGTCAGg	iron-regulated protein A (Vibrio)
Jann_2132	*hmuR-Smc01514-exbBD-tonB*	-143	4.28	gtTGACgcTTTAGTCgGA	hemin receptor, conserved protein, components of TonB-dependent transporters
Jann_2133	*hmuSTUV*	-132	4.28	TCcGACTAAAagcGTCAac	hemin ABC transporter
***Rhodobacterales bacterium* HTCC2654**					
RB2654_08847	*X-hmuP-hmuX**	-90	5	TCcGACTgAATTtGTCAGA	COG4256: hemin uptake protein, hypothetical conserved protein
RB2654_04329	*fbpA*	-53	4.96	cCTGACgAATTTAGTCAGA	ferric cations ABC transporter
RB2654_09494	*fecB-mxcB*	-66	4.74	cCcGACTgATTTAGTCAat	iron(III) dicitrate-binding protein, siderophore-interacting protein
		-33	4.55	TCcGACTgAAacAcTCAac	
RB2654_09499	*fecCDE*	-88	4.55	gtTGAgTgtTTcAGTCgGA	iron(III) dicitrate ABC transporter, permease subunits, ATPase
		-55	4.74	atTGACTAAATcAGTCgGg	
RB2654_15781	*hmuR*	-111	4.48	gtTGACTAAATTAcTaAGc	hemin receptor
RB2654_15786	*hmuSTUV-exbBD-X-tonB*	-119	4.48	gCTtAgTAATTTAGTCAac	hemin ABC transporter
RB2654_13154	*irpA*	9	4.48	TCcGACcAAATcAaTCAGc	iron-regulated protein A
***Roseobacter* sp. MED193**					
MED193_19679	*hmuP-hmuX*-irpA*	-117	5.1	TCTGACaAAAaTAGTCAGA	hemin uptake protein, hypothetical conserved protein, iron-regulated protein A
		-90	4.5	ctTGAtTgtTTTAaTCAGg	
MED193_15812		-140	5.1	TCTGACTAtTTTtGTCAGA	hypothetical protein A
MED193_19654	*bfd-bfr*	-102	4.25	cCTGACaAAAacAaTaAGc	bacterioferritin ferredoxin, bacterioferritin
		-71	4.37	cCTtAtTgtTTTtGTCAGA	
MED193_04321	*iscR -sufSBCD*	-143	3.96	ctTGACgAATTccaTCAGc	Fe-S cluster assembly
MED193_17499	*hmuSTUV*	-120	3.95	ggTGAtaAAAacAGTCAac	hemin ABC transporter
MED193_17504	*hmuR-Smc01514-exbBD-tonB*	68	3.95	gtTGACTgtTTTtaTCAcc	hemin receptor, conserved protein, components of TonB-dependent transporters
MED193_22076	*fecBCDE-mxcB*	-354	3.96	ctTGACaAAAcctGTaAGg	iron(III) dicitrate ABC transporter
***Roseovarius* sp. 217**					
ROS217_23057	*fbpA*	-97	4.9	TCcGACTAAAactGTCAGA	ferric cations ABC transporter
		-71	4.62	atTGACTctTTTAGTCAGA	
ROS217_22882	*fhuABCD*	-31	4.55	cCTGAgTtAAagAGTCgGA	ferrichrome iron receptor, ABC transporter

ROS217_10107	irpA	-146	4.43	gCctACTAAATcAGTCAac	iron-regulated protein A (Vibrio)
ROS217_17432	hmuSTUV	-112	4.23	TaTGAgTAAAagAGTCAag	hemin ABC transporter
ROS217_17442	hmuR	-199	4.23	ctTGACTctTTTAcTCAtA	hemin receptor
ROS217_23865	hmuP-hmuX*-bfd-bfr	-151	4.13	TCTGAtcAAAagAaTCgGA	hemin uptake protein, hypothetical conserved protein, ferredoxin, bacterioferritin
		-123	4.55	cCTtAtTAATTaAGTCAGA	
ROS217_20592	fbpC	-35	<u>4.07</u>	cCcGAtagATTTgaTCgGA	ferric cations ABC transporter, ATPase component
ROS217_22872	mxcB-Smc01514-exbBD-tonB	-56	<u>4.07</u>	gtTGACaAATTacaTCAGg	siderophore-interacting protein, components of TonB-dependent transporters
ROS217_22877	araX	-166	<u>4.07</u>	cCTGAtgtAATTtGTCAac	siderophore uptake AraC-type regulator
***Roseovarius nubinhibens* ISM**					
ISM_05125	fbpA	-139	5.11	CtTGACTAAATTAGTCgGa	ferric cations ABC transporter
		-105	4.95	tCcGAgTAtTTTAGTCgGa	
ISM_01000	hmuP-bfd-bfr	-204	4.98	tCTGACTAAAaaAGTCgGa	COG4256: hemin uptake protein, bacterioferritin ferredoxin, bacterioferritin
		-177	4.65	tCTGACagAATaAaTCAGa	
ISM_15940	ISM_15940-15945	-82	4.76	tCcGACTctTTTtGTCAGa	hypothetical protein, hypothetical protein A
ISM_04365	irpA	-81	4.72	CCcGAgTAtTTcAcTCAGG	iron-regulated protein A (Vibrio)
ISM_00940	OMP1	-116	4.67	CCTGAgTAAAagAcTCAGG	TonB-dependent siderophore receptor
ISM_04230	hmuSTUV	-109	4.28	tCaGACTAAAactGTCAaG	hemin ABC transporter
ISM_04235	hmuR	-158	4.28	CtTGACagtTTTAGTCtGa	hemin receptor
ISM_05670	OMP2	-99	<u>4.07</u>	CtTGACTttTTgccTCAGG	TonB-dependent siderophore receptor
***Loktanella vestfoldensis* SKA53**					
SKA53_06277	6277-dps	-32	4.86	gCTGACTAtATTtGTCGGg	COG0783, DNA-binding ferritin-like protein Dps
SKA53_14131	hmuP-hmuX*	-42	4.86	cCTGACTAAAaaAcTCAGg	COG4256: hemin uptake protein, hypothetical conserved protein
SKA53_06267	Jann3275	-48	4.64	gCTGACagAAaTAaTCAGA	hypothetical Fe-S protein
SKA53_05313	fbpA	-79	4.6	cCCGACTAAAaTAtTCAGg	ferric cations ABC transporter
SKA53_02241	fbpB	-73	4.5	TCCGAtcAAATaAGTCAGA	ferric cations ABC transporter
SKA53_06417	hmuR-Smc01514-exbBD-tonB	-112	4.47	ctTGACTgtTTgtGTCGGA	hemin receptor,conserved protein, components of TonB-dependent transporters
SKA53_06422	hmuSTUV	-121	4.47	TCCGACacAAacAGTCAag	hemin ABC transporter
SKA53_05183	iscR-sufSBCD	-130	<u>4.16</u>	ctTGACgAAAactaTCAGg	Fe-S cluster assembly
***Sulfitobacter* sp. EE-36**					
EE36_08018	araX	-114	4.96	TCCGAgTAAATTtGTCAGA	siderophore uptake AraC-type regulator
EE36_05618	fbpB	-27	4.94	TCCGACTgAATTAGTCGaA	ferric cations ABC transporter, permease component
EE36_10464	hmuP-hmuX*	-273	4.81	TCCGACTAAAactGTCGGA	COG4256: hemin uptake protein, hypothetical conserved protein
		-246	<u>4.02</u>	cCCtAtaAtTTcAaTCAGg	
EE36_01780	fhuA2-X-mxcB	-29	4.69	TCCGACTAAAacAGTaAGg	TonB-dependent siderophore receptor,siderophore-interacting protein
EE36_02713	irpA	-139	4.62	TCTtACTAAAaTtGTCGGg	iron-regulated protein A
EE36_08023	fhuABCD	-45	4.42	aCTGAgTtAAaTAcTCGGA	ferrichrome iron receptor, ABC transporter
EE36_12993	Smc01514-exbBD-tonB	-168	4.33	gtTGAtTgtTaaAGTCAGg	conserved protein, components of TonB-dependent transporters
EE36_05613	fbpA	-167	4.27	TtTGACacAAagAcTCAGg	ferric cations ABC transporter, periplasmic component
		-92	4.26	TCCtAtTAATTcAcTCAag	
EE36_01530	hmuR	-113	4.23	aCCGACTAAAagtGTCAac	hemin receptor
EE36_01535	hmuSTUV	-257	4.23	gtTGACactTTTAGTCGGt	hemin ABC transporter
EE36_10454	OMP1	-81	4.23	ctTGAtTgtTTTtaTCAGc	TonB-dependent siderophore receptor

EE36_16412	*fbpC*	-31	<u>3.98</u>	cCCGAgcgAATTgaTCGGA	ferric cations ABC transporter, ATPase component
***Oceanicola batsensis* HTCC2597**					
OB2597_05425	*hmuP-Smc01514-exbBD-tonB*	-193	4.53	ctTGACTAATTTgaTCGGA	COG4256: hemin uptake protein,components of TonB-dependent transporters
OB2597_08064	*OMP2*	-167	4.54	TCCGACTgATaTAGTCAcc	TonB-dependent siderophore receptor
OB2597_04193	*fbpA*	-61	4.66	cCTGACcAAAaTAcTCGGA	ferric cations ABC transporter, periplasmic component
OB2597_00230	*nuoABCDEFGHIJLMN*	-204	4.41	cCCtACTgAAagAGTCAGg	NADH ubiquinone oxidoreductase
OB2597_09404	*fecBCDE-mxcB*	-153	4.14	atCGACTgtTTTtGTCGGt	iron(III) dicitrate ABC transporter, siderophore-interacting protein
		-50	4.24	TCCGACTgAAacAaTCAtg	
OB2597_09409	*fhuA*	-391	4.24	caTGAtTgtTTcAGTCGGA	ferrichrome iron receptor
		-288	4.14	aCCGACaAAAacAGTCGat	
OB2597_06825	*hmuX**	-96	4.21	ggTGAgcAAAaTAGTCAGg	hypothetical conserved protein
OB2597_05460	*OMP1*	-100	4.1	TCTGACggtTTTtaTCAac	TonB-dependent siderophore receptor
OB2597_03589	*iscR -sufSBCD*	-133	<u>3.92</u>	CtTGACgacTTtgGTCAaG	Fe-S cluster assembly
OB2597_00425	*fbpC*	-27	<u>4.02</u>	cCTtAgTAAATccaTCgGA	ferric cations ABC transporter, ATPase component

<u>Color code for functional roles of genes:</u>

Genes involved in iron uptake

Genes involved in iron storage

Genes involved in iron utilization pathways (synthesis of Fe-S, heme; iron-containing enzymes)

Transcription factors (rirA, araX, fecIR, irr)

Genes with other or uncertain functional roles
