# Supplementary material for: Computational Reconstruction of Iron- and Manganese-Responsive Transcriptional Networks in α-Proteobacteria
Source: PLoS Comput Biol. 2006 Dec 15;2(12):e163. doi: 10.1371/journal.pcbi.0020163 (PMC1698941; doi:10.1371/journal.pcbi.0020163)
Supplement: Supplementary file 5 [file pcbi.0020163.st003.pdf]

Suppl. Table S3. The Irr regulon in alpha-proteobacteria

Locus ID	Operon	Position	Score	Irr site (ICE)	Function
Sinerhizobium meliloti					
SMc00529	*sufS2-sufBCDS1XA*	-132	6.02	AcTTTAGAAccgTTCaAAACT	Fe-S cluster assembly
SMc01119	*fssA*	-61	5.87	AtTTTgGAAcAATTCTAAAaa	hypothetical Fe-S scaffold protein
SMc03104	*hemA*	-245	5.87	AtTTTgGAATggTTCaAAACT	heme biosynthesis
SMc00784	*fbpA*	-147	5.76	AGTTTgGAAcAATTCcAAAaa	ABC-type Fe3+ transport system, periplasmic component
SMc00785	*rirA*	-73	5.76	ttTTTgGAATTgTTCcAAACT	Iron-responsive regulator RirA
SMc00359	*mbfA*	-32	5.67	taaTTAGAATTATTCTAAAga	membrane-bound ferritin
SMc04019	*hemH*	-75	5.29	ttTTTAGAAggATTtgAAACT	ferrochelatase (haem biosynthesis)
SMc00455		-99	5.28	cGTTTcGAACTGTTCgAAtCc	putative hemolysin III
SMc03787	*bfd-bfr*	-118	5.26	AGTTTAGAATcgcTCTAAtgc	bacterioferritin ferredoxin, bacterioferritin
SMb21432	*irp6A1-irp6BC*	-85	5.26	tGTTTgGAAgcgTTCTAAAag	ferrisiderophore ABC transporter
SMc00402	*irpA*	-117	4.86	AacTTcaAgccgTTCTAAACT	iron-regulated protein A (Vibrio)
Mesorhizobium loti					
mlr5523	*irpA- bfd-bfr*	-245	6.70	AGTTTAGAATAATTCTAAACT	iron-regulated protein A, bacterioferritin ferredoxin, bacterioferritin
mlr4601	*mbfA*	-36	6.63	AGTTTAGAAcAATTCTAAACT	membrane-bound ferritin
mlr0015	*sufS2-sufBCDS1XA*	-151	6.38	AGTTTAGAAcTATTCaAAACT	Fe-S cluster assembly
mll5315	*fssA*	-49	6.04	taTTTAGAAcTATTCcAAACT	hypothetical Fe-S scaffold protein
mlr4647		-33	5.20	AGcTTAGAAaTcTTCaAAACT	cytochrome b561, homologs in beta and gamma
mll2542		-89	5.16	ttTTTAtAATgATTCcAAAgT	probable drug transporter
mlr1147	*rirA*	-140	5.10	AGTcTgGAATTgTTCcAAggT	Iron-responsive regulator RirA
mll4333	*ccmABCDG*	-85	5.02	ttTcTgGAATAgTTCTAgACa	heme export
mlr6940	*katG*	-23	5.02	AGTcTgGAATcgTTCcAgAtT	catalase (peroxidase I)
***Mesorhizobium* sp. BNC1**					
MBNC03003400	*fbpAB*	-142	6.70	AGTTTAGAATAATTCTAAACT	ferric cations ABC transporter
MBNC03003799	*sufS2-sufBCDS1XA*	-136	6.31	AGTTTAGAAcTgTTCaAAACT	Fe-S cluster assembly
MBNC03003055	*mbfA*	-38	6.20	AGTTTAGAATTATTCTAAtCa	membrane-bound ferritin
MBNC03003089	*fssA*	-43	5.87	AtTTTAGAATTgTTCcAAAaa	hypothetical Fe-S scaffold protein
MBNC03002826	*katG*	-37	4.95	AGTcTgGAATAgTTCTAgggT	catalase (peroxidase I)
Rhizobium leguminosarum					
RL2583	*sufS2-sufBCDS1XA*	-132	6.36	AGTTTAGAAcAATTCgAAACT	Fe-S cluster assembly
RL0263	*mbfA*	-35	6.06	gaTTTAGAATTATTCTAAAaT	membrane-bound ferritin
RL0777	*rirA*	-68	5.95	AtTTTgGAATTgTTCaAAACT	Iron-responsive regulator RirA
RL0400	*fssA*	-43	5.30	tcTTTtGAAgcATTCTAAAga	hypothetical Fe-S scaffold protein
RL1432	feuPQ-lipA-cycHJKL	-108	5.30	AGTcgAGAAcAgTTCTAAACa	regulator, maturation of cytochrome c?
RL4379	*hemA1*	-113	5.36	taTcTgGAATcATTCaAAACT	heme biosynthesis
RL0322	*irpA*	-205	5.24	ttcTTAaAATcATTCcAAACT	iron-regulated protein A
RL2703	*fumA*	-55	4.91	gaTTTAGAccggTTCcAAtCT	fumarate hydratase class I
RL2713	*irp6ABC*	-93	4.79	AGTTTgGAAacgcTCTAAAac	ferrisiderophore ABC transporter
Exprerimental data:	***Irr/iron-regulated genes (Todd et al., 2006b)***				

**Rhizobium etli**					
RHE_CH00257	*mbfA*	-35	6.22	gGTTTAGAATTATTCTAAAtT	membrane-bound ferritin
RHE_CH00304	*irpA*	-98	5.49	AtcTTAaAATTATTCcAAACT	iron-regulated protein A
RHE_CH00381	*fssA*	-56	5.38	tcTTTtGAAgAATTCTAAAga	hypothetical Fe-S scaffold protein
RHE_CH00735	*rirA*	-70	5.95	AtTTTgGAATTgTTCaAAACT	Iron-responsive regulator RirA
RHE_CH02254	*sufS2-sufBCDS1XA*	-205	6.36	AGTTTAGAAcAATTCgAAACT	Fe-S cluster assembly
RHE_CH03846	*hemA1*	-117	5.18	tGTcTgGAATcATTCaAAAaa	heme biosynthesis
RHE_CH02385	*fumA*	-157	4.55	caTTTAGAtcggTTCcAAttT	fumarate hydratase class I
**Agrobacterium tumefaciens**					
AGR_C_3350	*sufS2-sufBCDS1XA*	32	6.43	AGTTTAGAATAATTCgAAACT	Fe-S cluster assembly
AGR_C_430	*mbfA*	-80	5.24	ttgTTAGAATTATTCTAAAac	membrane-bound ferritin
		-33	6.05	taTTTAGAATTATTCTAAAaa	
AGR_C_344	*rirA*	-65	5.61	ttTcTgGAATTgTTCTAAACT	Iron-responsive regulator RirA
AGR_C_346	*fbpA*	-84	5.61	AGTTTAGAAcAATTCcAgAaa	ABC-type Fe3+ transport system, periplasmic component
AGR_L_1858	*irgA*	-78	5.65	AaTTTAGAAcTgTTCgAAAac	iron-regulated outer membrane receptor irgA
AGR_C_616	*fssA*	-57	5.64	ttTTTgGAAgAATTCTAAAtT	hypothetical Fe-S scaffold protein
AGR_C_493	*irpA*	-40	5.45	tGTTTcaAAgcATTCTAAACT	iron-regulated protein A
AGR_L_1661	*fhuADBC*	-167	5.49	AGaTTtGAATAATTCcAAgCT	hydroxamate-dependent iron uptake
AGR_C_2495	*fdx*	-70	5.27	tGTTTAGAAcAATTCTcAgaT	ferredoxin, 2fe-2S
AGR_C_5028	*bfd-bfr*	-119	5.26	AGTTTAGAAcgAgTCTAgAga	bacterioferritin ferredoxin, bacterioferritin
AGR_C_4738	*hemA*	-62	5.20	ttaTTgGAATggTTCaAAACa	heme biosynthesis
AGR_C_4488	*fatBDCE*	-44	5.17	AaTTcAGAAccgTTCTAAAac	ferric anguibactin ABC transporter
AGR_L_2862	*irp6AABC*	-90	4.97	AGTTaAGAATggcTCTAAAac	ferrisiderophore ABC transporter
**Brucella melitensis**					
BMEI0354	*mbfA*	-204	6.39	AtTTTAGAATTATTCTAAAtT	membrane-bound ferritin
BMEII0885	*tpd -X- ftr1 -COG0348*	-136	5.81	tGTTTAGAATTgaTCTAAACT	High-affinity Fe2+ transport component and permease, polyferredoxin
BMEII0707	*rirA*	-161	5.41	ttTTaAGAATgATTCTAAAgT	Iron-responsive regulator RirA
BMEI1892		-306	5.70	AGTcTAGAATAtTTCTAAACT	hypothetical cytosolic protein (cysteine-rich)
BMEI1724		-102	5.18	tcTTTAaAgccATTCTAAAtT	hypothetcial protein
BMEII0705	*bfd-bfr*	-300	5.05	ttTTTAGAATAtTTCaAAAac	bacterioferritin ferredoxin, bacterioferritin
BMEI1042	*sufBCDS1X*	-118	4.75	AGTTTAGAgaggTTtTAAAga	Fe-S cluster assembly
**Bartonella quintana**					
BQ11660	*mbfA*	-37	5.97	AtcTTAGAATTATTCTAAAgT	ferritin-like and TM domains
BQ01080	*ccmABCDG*	-97	5.14	ttTcTAGAAcTATTCTAgAta	heme export
BQ05950	*sufBCDS1XA*	-149	5.14	gaaTTAGAATTATTtTAAAaa	Fe-S cluster assembly
**Bradyrhizobium japonicum**					
blr7895	*mbfA*	-39	5.95	AaTTTAGAATCATTCTAAACT	membrane-bound ferritin
bsl6680	*bfr*	-61	5.50	caTTTAGAAgCgTTCTAAAtT	bacterioferritin
bsl6681	*bfd*	-127	5.19	AtTTTAGAgcCgTTCTAggCT	bacterioferritin ferredoxin
bll7968	*fhuA3-piuB*	-115	5.12	cGcTTAGAAcCATTCaAcACT	outer membrane iron receptor, Iron-uptake factor
blr4339	*sufBCDS1XA*	-103	5.07	cGgcTtGAAcGgTTCTAAgCg	Fe-S cluster assembly
blr4504	*fhuA1 -blr4505*	-170	4.90	cGTcTAGAAgGATTCcAAAtc	outer membrane iron receptor

blr3904	*fiu-piuC-exbBD-tonB2*	-185	4.83	gtTTTAGAAcGAcTCcAAttT	outer membrane iron receptor, iron uptake factor, iron transporter components
		-163	4.81	AcTTTAGAAcCgTTtgAAACT	
		-140	4.98	AGaTTAGAATCgTTtTgAtCT	
bll5796	*fumA*	-61	4.83	AGcTTAGAAcGcTTCTAtgCc	fumarate hydratase, class I
blr0512	*sdhCDAB*	-267	4.79	gGcTTAGAgcCgTTCcAAtag	succinate dehydrogenase
bsr6522	*feoAB*	-80	4.64	AcTTTAaAATCAcTCTAAggT	ferrous iron transporter
blr0778	*katG*	113	4.64	gGcTTAGAgTGgTTCTAcctg	catalase (peroxidase I)
bll0768	*irr*	-95	4.63	cGaTTAGAAcCtcTCTAgttg	iron response regulator
bll7076	*hmuR-XX-exbBD-tonB1*	-166	4.62	AaTTTAcAATCgaTaTAAACT	hemin receptor
blr7077	*hmuTUV*	-303	4.62	AGTTTAtAtcGATTgTAAAtT	hemin ABC transporter
bll3138	*nuoEF-fdhFD-bll3134*	-57	4.57	AtcTTcGAATGATTCcAAAta	NAD-dependent formate dehydrogenase
bll2737	*bll2737-bll2736*	-51	4.56	tGaTTtGAAcagTTCcAAttT	Fe-S oxidoreductase, putative aldehyde dehydrogenase
blr674	*blr6742-blr6743-blr6744*	-126	4.51	AcTTcgGAAgAgTTCTAAtaa	pyridine nucleotide-disulphide oxidoreductase, ferredoxin oxidoreductase
blr2933		-45	4.44	tGaTTgGAATGATTtcAgACT	probable aerotaxis receptor
bll4919	*nuoABCDEFGHIJLMN*	-68	4.43	gGcTcAGAATaATTCcAAAtc	NADH ubiquinone oxidoreductase
bll4920	*fhuA2*	-120	4.43	AtgTTgGAtcGcTTCTAAgag	outer membrane iron receptor
blr1720	*hupSLCDFGHIJK-hypABFCD*	-82	4.43	AcTTTtGAATCgcTCcAggCT	membrane-bound uptake hydrogenase
bll0466	*acnA*	-134	4.41	cGgTTAGAAaGcTTCTAtAag	aconitase
bll5701	*cycB-bll5700*	-101	4.22	AaTTTtGAAcGATTCaAtttc	cytochrome b561, glutathione S-transferase
Expression data:	***Iron- and/or Irr-regulated genes (Roudolph et al., 2006 and Yang et al., 2006)***				
***Bradyrhizobium* sp. BTAi1**					
Brad_6124	*fhuA2-piuB2*	-112	4.36	gagTTttAATAATTCTAAAta	outer membrane iron receptor, Iron-uptake factor
Brad_3189	*mbfA*	-59	5.86	AcTTTAGAAgcgTTCTAAACa	membrane-bound ferritin
Brad_5789		-35	5.16	ttTTTAGAAcgtTTCTAAttT	conserved hypothetical protein
Brad_3567	Brad_3567 - *fhuA-piuB*	-151	5.07	gaTTTgGAAccgTTCTAAtaa	outer membrane iron receptor, Iron-uptake factor
Brad_3512	*feoAB*	-78	5.02	tGTTTAaAATcgcTCTAAgaT	ferrous iron transporter
Brad_2353		-77	4.97	AaTcTAGAAgcATTCcAAtCa	probable aerotaxis receptor
Brad_4228	*fiu-piuC-exbBD-tonB2*	-198	4.86	gtTTTAGAAcgATTtcAAgtT	outer membrane iron receptor, iron uptake factor, iron transporter components
Brad_4228		-156	4.88	ttTTTAGAATTgTTtTgAtCT	
Brad_0131		-106	4.80	AacTcAGAAcAgTTCaAAAaa	regulatory protein, similar to LuxR
Brad_1683	*bfd-bfr*	-95	4.38	AGaTTgGAATAgcTTcAAAag	bacterioferritin ferredoxin, bacterioferritin
Brad_5162	*irr*	-76	4.25	AGTTTAGgggcgTTCTAgAtc	iron response regulator
Brad_3677	*nifHDKX*	-201	4.25	ccTTTttAAcgATTCcAAAgc	molybdenum-iron nitrogenase
Brad_1627	*Brad_1627-1626-1625*	-175	4.21	gccTTgGAATggcTCTAgtCT	pyridine nucleotide-disulphide oxidoreductase, ferredoxin oxidoreductase
Brad_3993	*nuoEF-fdhFD-bll3134*	-62	4.14	taTcTtGAgcAATTCcAAttc	NAD-dependent formate dehydrogenase
Brad_3670	*frd-sufBCDS*	-109	4.13	AaTTTAGAATccTcCTtAgaa	ferredoxin, FeS cluster assembly
***Rhodopseudomonas palustris* CGA009**					
RPA4303	*mbfA*	-33	5.51	taTTTAGAAgCgTTCTAAACT	membrane-bound ferritin
RPA4636	*feoAB*	-78	5.19	ctgTTAcAATCgTTCTAAACT	ferrous iron transporter
RPA1876	*fhuA1*	-222	4.95	tGaTTtGAAcGATTCaAAAaT	outer membrane iron receptor
RPA4152	*fbpAB*	-99	4.81	cGgcTgGAATagTTCTAAtCg	ferric cations ABC transporter

RPA3600	bfr	-66	4.79	gaTcTAGAATtATTCTAAttc	bacterioferritin
RPA0765	RPA0765	-317	4.74	gGTTTtGAAgGccTCTAAtCg	outer membrane iron receptor
RPA1845	fcuA3	-266	4.73	AGcTcgGAATCgTTCTAgAtT	outer membrane iron receptor
		-129	4.43	AcaaTAGAgTCgTTCTAAgtT	
RPA3195	iorAB	-73	4.60	AGcTTAGAcTGgTTCgAAACa	indolepyruvate ferredoxin oxidoreductase
RPA3480	fiu-piuC-exbBD-tonB3	-306	4.60	ttcTTAGAATGATTtTgAtCT	outer membrane iron receptor, iron uptake factor, iron transporter components
RPA2464	sufBCDS1XA	-101	4.58	gtgcTtGAAcGgTTCTAAgag	Fe-S cluster assembly
RPA1224	iorAB2-oorABC	-51	4.47	ccTTTAcAgcagTTCTAAtCg	indolepyruvate ferredoxin oxidoreductase, 2-oxoglutarate dehydrogenase
RPA4430	RPA4430-piuB	-87	4.47	ccgTTAGAcTGATTCcAAgCT	outer membrane iron receptor, iron-uptake factor
RPA2307	RPA2307	-207	4.46	AGacTAGAATCtcTCaAgACT	outer membrane iron receptor
RPA2308	RPA2308-2310	-48	4.46	AGTcTtGAgaGATTCTAgtCT	hypothetical iron siderophore ABC transporter
RPA3282	fecIR -hmuR2	-112	4.45	cGgcTAGAgcCAaTCTAAttc	iron transport sigma factor and sensor, heme receptor
RPA2026	fcuA1	-123	4.29	tcgcTAGAAcGgTTCTAgtgg	ferrichrome receptor
RPA0489	fdx	-26	4.21	AaaTTAGAAcCgTTCcggAgc	ferredoxin II
RPA3414	fhuA2-X	-115	4.18	tGTTTcGAgcaATTCcAAtCc	outer membrane iron receptor
RPA2121	hmuPTUV	-86	4.14	cGTTTAtAgTaATTaTAAgac	heme ABC transporter
RPA2122	hemN3	-267	4.14	gtcTTAtAATtAcTaTAAACg	paralog of oxygen-independent coproporphyrinogen III oxidase
RPA3840	fhuA3-XX	-110	4.13	gacgTAGAAcGtTTgTAAtCg	outer membrane iron receptor
RPA2124	hmuR1-XX-exbBD-tonB1	-163	4.04	AagTTAtAATtgcTtTAAtCa	heme uptake
RPA3876	fumA	-81	3.94	cccTTAGAggtATaCTAAAtg	fumarate hydratase class I
RPA2390	rhbCF-XXX-fatBDCE-fecR	-112	3.91	tGaTTgGttgaATTCTAAACT	siderophore biosynthesis, ferric anguibactin ABC transporter
**Nitrobacter winogradskyi Nb-255**					
Nwi_2475	bfr	-80	5.15	gaTTTAGAAgtATTCTAAAtT	bacterioferritin
Nwi_0035	irr	-113	4.87	AGTTTtGAgcCATTCTAgttg	iron regulatory protein Irr
Nwi_3045	fecIR - fhuA1	-62	4.76	AGcTTAGAAcCAaTCcAgACa	iron sigma factor and sensor, outer membrane iron receptor
Nwi_0894	fecIR - omp	-226	4.66	tcTcTAGAAcCAaTCTAAAtg	iron sigma factor and sensor, outer membrane iron receptor
Nwi_1340	fecIR - fhuA3	-55	4.6	gGTTTAGAtgCgaTCTAAAgT	iron sigma factor and sensor, outer membrane iron receptor
Nwi_2582	cycA1	-169	4.52	AcacTcGAATCgTTCTAAgaT	cytochrome c class I
Nwi_1204	glgP	-198	4.51	cGcTTAaAAaCgcTCTAAgtg	glycogen phosphorylase
Nwi_2048	fecI1	-139	4.45	cacTTAGAAgCtcTCcAAtCT	iron transport sigma factor
Nwi_2462		-140	4.45	gGTTTAtAgcGtTTCaAAACg	hypothetical protein
Nwi_1467		-94	4.41	gagaTAGAATCATTCcAAcCg	hypothetical protein
Nwi_0773	cycA1-narG	-144	4.36	gtTTTctAAcGATTCTAgtCT	cytochrome c class I, nitrate reductase
Nwi_0902	fecI2	-88	4.36	ctcTTAaAccCATTCTAAAtc	iron transport sigma factor
Nwi_2813	piuB -COG3656	-128	4.35	taTTTAGAATGAacCTAAtCT	Iron-uptake factor, hypothetical lipoprotein
Nwi_1734	fecIR - omp	-21	4.31	ttccTAGAATCAaTCTAgAtc	iron sigma factor and sensor, outer membrane iron receptor
Nwi_2610		-81	4.3	cGgTTAGAAaagTTCaAgttg	hypothetical protein
Nwi_0703	exbBD-tonB2	-474	4.26	AaTcTAGAAgGATTCTtAAgT	components for TonB-dependent iron transporters
Nwi_0700	fiu-piuC	-92	4.25	gGTTTAGAtgaATTCTAtAaT	outer membrane iron receptor, iron uptake factor piuC
Nwi_2883	fecIR - fhuA2	-196	4.19	ttcTTAaAcTCATTCTAAAta	iron sigma factor and sensor, outer membrane iron receptor

Nwi_3079	*fecIR - fhuA4*	-249	4.17	ttTcTAGAAcaggTCTAAAtc	iron sigma factor and sensor, outer membrane iron receptor
Nwi_1966	*fecI3*	-562	4.06	AcTTTgGAccGgaTCTAAAtc	iron sigma factor
Nwi_0724	*fecR -fhuA5*	-45	3.93	cGcTTgaAAaCgcTCTAggaT	iron sensor, outer membrane iron receptor
Nwi_2053	*tonB*	-279	3.91	AcTTTcGAAgtATTCaAgttT	component for TonB-dependent iron transporters
Nwi_2051	*fecIR - omp*	-126	3.89	gcaTTAGAcgCtaTCTAAACT	iron sigma factor and sensor, outer membrane iron receptor
Nwi_0242	*hemA*	-65	3.86	ccgcTgGAAaGgcTCTAAAaa	heme biosynthesis
Nwi_2976	*feoAB*	-197	3.85	AcTTTttAATCATTCcAgcaa	ferrous iron transporter
***Nitrobacter hamburgensis* X14**					
Nham_3279	*fiu2-piuC2*	-245	5.13	AaTTTAGAATCgTTtTAAAaa	outer membrane iron receptor, iron uptake factor piuC
		-152	4.31	gaTTTAGAtaGATTCaAAAaa	
Nham_3277	*exbBD-tonB*	2	5.15	tGTTTAGAAcGcTTCTAAtaa	components for TonB-dependent iron transporters
Nham_3901	*bfr*	-95	5.05	gaTTTAGAAgCATTCTAAgaa	bacterioferritin
Nham_3555	*fhuA*	-177	4.96	AaTTTAGAAcGATTaaAAAtg	outer membrane iron receptor
		-124	4.27	AaTaTtGAtctATTCTAAACg	
Nham_2133	*feoAB*	-61	4.85	taTTTttAAcCATTCTAAtCT	ferrous iron transporter
Nham_1013	*irr*	-105	4.83	AGTTTgGAgcCgTTCTAgttg	iron regulatory protein Irr
Nham_3637	*cycA1*	-164	4.63	AtgcTcGAATCATTCTAAgaT	cytochrome c class I
Nham_2257		-90	4.55	cagcTAGAATCATTCcAgttT	hypothetical lipoprotein
Nham_4206	*cycB*	-121	4.48	ctcTTAGAtgGATTCcAAtCa	cytochrome b561
Nham_1606	*piuB2 -COG3656*	-148	4.41	AGTTTAGAgcGAacCTAAtCT	Iron-uptake factor, hypothetical lipoprotein
Nham_1610	*fiu1-piuC1*	-138	4.17	gagcTAGAAcCtcTgTAAACT	outer membrane iron receptor, iron uptake factor piuC
Nham_1611	*exbBD-tonB2*	-125	4.33	gtTTTAaAgaGATTCTAAAac	components for TonB-dependent iron transporters
Nham_2255	*fcuA1-piuB*	-102	4.32	gacTTAGAtcGATTCTActtc	outer membrane iron receptor, Iron-uptake factor
Nham_1436	*cycA2-narG*	-143	4.22	gcTTTctAAcGATTCTAgtCT	cytochrome c class I, nitrate reductase
Nham_4406	*hemA*	-67	4.19	cGgcTgGAAaGgcTCTAAAaa	heme biosynthesis
Rhodobacter sphaeroides					
RSP_0850	*mbfA*	-30	5.72	AaTTTAGAATGAcTCTAAAaa	membrane-bound ferritin
RSP_2395	*ccpA*	-86	4.81	AGaTTAGAATtgaTCTAAgtc	cytochrome c peroxidase
Rhodobacter capsulatus					
RRC04069	*mbfA*	-28	5.55	AtgTTgGAATCATTCTAAACT	membrane-bound ferritin
RRC03475	*ccpA*	-44	5.15	tGTTTAGAAgtATTCcAtAtT	cytochrome c peroxidase
Silicibacter pomeroyi					
SPO3842	*mbfA*	-33	5.68	AtcTTgGAATCATTCTAAAtT	membrane-bound ferritin
SPO0382	*fssA*	-75	5.62	tcTTTAGAAcCgTTCcAAAgT	hypothetical Fe-S scaffold protein
SPO2025	*iscR-sufSBCD*	-120	5.19	AtcTTAGAATGgTTCTAAgtc	Fe-S cluster assembly
SPO0330	*ccpA*	-73	5.11	AtgTTAGAATGATTCTAAtCg	cytochrome c peroxidase
***Silicibacter* sp. TM1040**					
TM1040_3091	*fssA*	-75	5.64	AaTTTAGAAccATTCcAAAac	hypothetical Fe-S scaffold protein
TM1040_1240	*iscR-sufSBCD*	-118	5.61	AccTTAGAActgTTCTAAAga	Fe-S cluster assembly
TM1040_2791	*katG*	-145	5.52	AacTTAGAATAATTCTgAACT	Catalase (peroxidase I)
***Jannaschia* sp. CC51**					

Jann_0865	*fssA*	-78	4.84	AcTTTAGAAcgATTCcAgttc	hypothetical Fe-S scaffold protein
Jann_3276	*dps*	-124	6.11	AGTTTAGAATTATTCTAgACa	DNA-binding ferritin-like protein Dps
Jann_2366	*iscR-sufSBCD*	-136	5.14	AcccTAGAAcgATTCcAAAtT	Fe-S cluster assembly
***Rhodobacterales bacterium* HTCC2654**					
RB2654_18613	*fssA*	-53	4.81	gacTTgGAAcgATTCcAAgCT	hypothetical Fe-S scaffold protein
RB2654_16991	*mbfA*	-31	5.84	AGTTTgGAATcgTTCcAAACa	membrane-bound ferritin
RB2654_13154	*irpA- bfd-bfr*	-45	5.36	tGaTTgGAAccATTCcAAAtT	iron-regulated protein A (Vibrio),bacterioferritin ferredoxin, bacterioferritin
***Roseobacter* sp. MED193**					
MED193_04321	*iscR-sufSBCD*	-123	5.25	AtcTTAGAAcAATTCTAAttg	Fe-S cluster assembly
MED193_04676	*ahpC*	-58	5.3	AaTTTAGAAcTATTaTAAAtg	peroxiredoxin/glutaredoxin family protein
MED193_10358	*fssA*	-76	6.22	AaTTTAGAATAATTCTAAACc	hypothetical Fe-S scaffold protein
***Roseovarius* sp. 217**					
ROS217_20542	*iscR-sufSBCD*	-99	5.84	AccTTAGAAcTgTTCTAAAtT	Fe-S cluster assembly
ROS217_10487	*fssA*	-77	5.7	AtcTTAGAATggTTCTAAACc	hypothetical Fe-S scaffold protein
ROS217_11126	*mbfA*	-31	6.16	AGTTTAGAATTATTCTAAAag	membrane-bound ferritin
ROS217_11501	*ccpA*	-76	5.42	gaTTTAGAATggTTCcAAACg	cytochrome c peroxidase
***Roseovarius nubinhibens* ISM**					
ISM_02850	*fssA*	-76	5.47	ttTTTAGAATcgTTCcAAAtc	hypothetical Fe-S scaffold protein
ISM_08740	*mbfA*	-38	5.76	AGTTTAGAATcATTCTAAttg	membrane-bound ferritin
ISM_16015	*iscR-sufSBCD*	-128	6.18	AcTTTAGAAcTgTTCTAAACa	Fe-S cluster assembly
ISM_16225	*katG*	-93	4.64	cGcTTAGAAcgATTCcAAggc	catalase (peroxidase I)
***Loktanella vestfoldensis* SKA53**					
SKA53_14166	*mbfA*	-33	5.97	AGaTTAGAATcATTCTAAAta	membrane-bound ferritin
SKA53_14701	*fssA*	-77	5.8	AGTTTAGAATcgTTCcAAAtc	hypothetical Fe-S scaffold protein
SKA53_05183	*iscR-sufSBCD*	-110	5.74	AccTTAGAAggATTCTAAACT	Fe-S cluster assembly
SKA53_02751	*hemA*	-63	4.14	AGaTTAGAAcgATTCatggCg	heme biosynthesis
SKA53_14466	*katG*	-84	4.77	AtgTTAGAAggATTCTAAgCg	catalase (peroxidase I)
***Sulfitobacter* sp. EE-36**					
EE36_14302	*iscR-sufSBCD*	-117	5.69	AGTTTAGAAcggTTCTAActT	Fe-S cluster assembly
EE36_14417	*hemA*	-102	4.73	tGaaTtGAATggcTCTAAACT	heme biosynthesis
EE36_01790	*dps*	-117	6.1	AGTTTAGAATAATTCcAAAaa	DNA-binding ferritin-like protein Dps
EE36_09510	*fssA*	-75	5.44	AacTTAGAATcgTTCcAAACc	hypothetical Fe-S scaffold protein
EE36_11399	*katG*	-111	4.43	ctgTTAGAAcAATTCcAgAaa	catalase (peroxidase I)
***Oceanicola batsensis* HTCC2597**					
OB2597_06815	*bfr*	-84	5.95	ttTTTgGAATAATTCTAAAtT	bacterioferritin
OB2597_10089	*fssA*	-80	5.19	gaaTTAGAAcggTTCTAAAtc	hypothetical Fe-S scaffold protein
OB2597_01932	*hemA*	-92	4.21	gacTTAaAAccATTCcAAttc	heme biosynthesis
OB2597_03589	*iscR-sufSBCD*	-113	4.93	AccTTAGAAagATTCTAAAgc	Fe-S cluster assembly
OB2597_11886	*katG*	-64	4.25	gtgTTAGAtTgATTCTAAAgg	catalase (peroxidase I)
***Pelagibacter ubique* HTCC1002**					
PU1002_04356	*rbr*	-76	5.74	AcaTTAaAATAATTCTAAACT	Rubrerythrin

PU1002_04361	*irr*	-23	5.88	AGTTTAGAATTATTaTAAACa	iron regulatory protein
Rhodospirillum rubrum ATCC11170					
Rru_A0038	*ccmCDE*	-161	5.46	TTTCTgGAAgAcTTCcAGAtA	heme export
Rru_A1789	*ccpA*	-97	4.61	TTcCTAGAgcAATTCcAatAt	cytochrome c peroxidase
Magnetospirillum magneticum AMB-1					
amb2822	*ccpA*	-163	5.24	gagtTAGAAgAATTCTAaAtA	cytochrome c peroxidase
Magnetospirillum magnetotacticum MS-1					
Magn03010382	*rbr*	-112	5.02	aaTCTcGAATTAaTCgAtAAA	Rubrerythrin

Color code for functional roles of genes:

Genes involved in iron uptake

Genes involved in iron storage

Genes involved in iron utilization pathways (synthesis of Fe-S, heme; iron-containing enzymes)

Transcription factors (rirA, araX, fecIR, irr)

Genes with other or uncertain functional roles
