# Supplementary material for: Computational Reconstruction of Iron- and Manganese-Responsive Transcriptional Networks in α-Proteobacteria
Source: PLoS Comput Biol. 2006 Dec 15;2(12):e163. doi: 10.1371/journal.pcbi.0020163 (PMC1698941; doi:10.1371/journal.pcbi.0020163)
Supplement: Supplementary file 6 [file pcbi.0020163.st004.pdf]

Suppl. Table S4. The Mur regulon in alpha-proteobacteria

Locus ID	Operon	Positio	Score	Site	Function
Sinerhizobium meliloti					
SMc02509	***sitABCD***	-81	5.38	gTTGCAAATGcTTctCAtT	Manganese ABC transporter
***Mesorhizobium sp.* BNC1**					
MBNC03003615	***mntH***	-39	5.22	AaTGCAAATaAgTcGCAAc	Manganese permease
MBNC03001420	***sitABCD***	-114	5.73	gTTGCAAATGgTTTGCAAT	Manganese ABC transporter
Rhizobium leguminosarum					
RL0940	***mntH***	-67	5.66	gTTGCgAATGAcTTGCAtT	Manganese permease
RL3884	***sitABCD***	-139	4.65	gTTGCAAtTCATTcttAAT	Manganese ABC transporter
		-85	5.37	tTTGCAAATCATTctgAAT	
Rhizobium etli					
RHE_CH00878	***mntH***	-67	5.66	gTTGCgAATGAcTTGCAtT	Manganese permease
Agrobacterium tumefaciens					
AGR_L_798	***sitABCD***	-77	6.01	ATTGagAATGATTTGCAAT	Manganese ABC transporter
		-25	4.84	ATTGCgAgTtAaTcGCAAc	
AGR_C_3186	***mntH***	9	5.66	taTGagAATGATTTGCAAT	Manganese permease
Brucella melitensis					
BMEI0569	***mntH***	-43	5.29	AaTGCAAATagTTTGCAAc	Manganese permease
BMEI1563	*irr1*	-201	5.90	ATTGCAAAcCATTTGCAAT	iron regulatory protein Irr
Bartonella quintana					
BQ00790	***sitABCD***	-34	5.82	AaTGCAAATCATTgGCAAT	Manganese ABC transporter
Bradyrhizobium japonicum					
blr5044	***mntH***	-45	5.74	gTTGCAAATGAgTTGCAAT	Manganese permease
bll0768	*irr1*	-78	4.34	gTTGCgAgaaAcTTGCAtc	iron regulatory protein Irr
***Bradyrhizobium* sp. BTAi1**					
Brad_4860	***mntH1***	-46	5.12	cTTGCAAATaAgTTGCAtT	Manganese permease
Rhodopseudomonas palustris					
RPA2706	***mntH***	-48	5.70	ATTGCAAATGAcTTGCAAg	Manganese permease
RPA0424	*irr1*	-84	4.33	gTTGCgAAgCgcTTGCAcc	iron regulatory protein Irr
Nitrobacter winogradskyi					
Nwi_1912	***mntH***	-52	5.7	tTTGCAAATGAgTTGCAAT	Manganese permease
Nwi_0035	*irr*	-96	4.6	gTTGCgAAaaAcTTGCAtc	iron regulatory protein Irr
Nitrobacter hamburgensis X14					
Nham_0774	***mntH***	-49	5.09	tTTGCAAgTGAgTTGCAAg	Manganese permease
Nham_1013	*irr*	-88	4.6	gTTGCgAAaaAcTTGCAtc	iron regulatory protein Irr
Rhodobacter sphaeroides					
RSP_0904	***sitABCD***	-8	5.22	tTTGCgAATGccTcGCAtT	Manganese ABC transporter
Silicibacter pomeroyi					
SPO3366	***sitABCD***	-83	5.28	cTTGCgAATaATTctCAAT	Manganese ABC transporter
		-52	5.00	gTTGCgAATGgTTctCAta	
SPOA0445	*irr*	-77	4.13	gaTGagAATGAaTTcCAtT	iron regulatory protein Irr
Silicibacter sp. TM1040					
TM1040_0098	***sitABCD***	-96	4.49	tgTGagAATaATTctCAtT	Manganese ABC transporter
		-65	4.68	gTTGCgAAgCgTTctCAta	
TM1040_0183	*irr*	-74	4.52	tTTGCAAATCcgTTtgAAa	iron regulatory protein Irr
Jannaschia sp. CC51					
Jann_2080	***sitABCD***	-87	5.01	tTTGCAAcTCAcTcGCAAg	Manganese ABC transporter
Jann_1136	*irr*	43	4.61	tTTGCAAAgaccTcGCAAa	iron regulatory protein Irr
Sulfitobacter sp. EE-36					
EE36_12418	***sitABCD***	-111	4.96	taTGCgAcTaATTcGCAtT	Manganese ABC transporter
Rhodobacterales bacterium HTCC2654					
RB2654_19543	***sitABCD***	-166	5.57	gTTGCAAATGATTTGCAcT	Manganese ABC transporter
		-118	5.31	gTTGCAAtTCATTcGCAAc	
Loktanella vestfoldensis SKA53					
SKA53_08116	***sitABCD***	-64	4.89	gTTGCgAAcGcTTctCAtT	Manganese ABC transporter

Roseobacter sp. MED193					
MED193_12968	***sitABCD***	-86	5.1	gTTGagAATaATTctCAtT	Manganese ABC transporter
		-55	4.78	gTTGagAATCgTTctCAta	
MED193_17849	*irr*	-41	4.94	AaTGCAAtTCATTcGCAgT	iron regulatory protein Irr
Roseovarius sp.217					
ROS217_05129	***sitABCD***	-27	5.11	gTTGCgAATaATTctCAta	Manganese ABC transporter
Roseovarius nubinhibens ISM					
ISM_02005	***mntX****	-30	5.63	ATTGagAATaATTcGCAAT	predicted Mn transporter
Oceanicola granulosus HTCC2516					
OG2516_13601	***mntX****	*-38*	5.85	ATTGCgAATtATTcGCAAT	predicted Mn transporter
Oceanicola batsensis HTCC2597					
OB2597_13568	***sitABCD***	-92	5.43	gTTGCgAATGgTTcGCAtT	Manganese ABC transporter
		-61	5.26	gTTGCgAATGgTTcGCAtc	
