# Supplementary material for: Computational Reconstruction of Iron- and Manganese-Responsive Transcriptional Networks in α-Proteobacteria
Source: PLoS Comput Biol. 2006 Dec 15;2(12):e163. doi: 10.1371/journal.pcbi.0020163 (PMC1698941; doi:10.1371/journal.pcbi.0020163)
Supplement: Supplementary file 7 [file pcbi.0020163.st005.pdf]

Suppl. Table S5. The MntR regulon in alpha-proteobacteria

Organism	Locus ID	Operon	Position	Score	Site	Function
Mesorhizobium loti	mlr2501	***mntH***	*-196*	5.98	TTTATAGCaCGGGCTcTgAt	NRAMP-type Mn transporter / mntR
***Mesorhizobium* sp. BNC1**	MBNC03003581	***mntR***	*-47*	5.79	TccATAGCCgaGGCTATAAt	mntR
Rhodobacter capsulatus	5_0455	***mntH***	*-48*	6.44	AATaTAGCCAaGGCTACAcT	NRAMP-type Mn transporter
***Magnetospirillum magnetotacticum* MS-1**	Magn03007105	***mntH***	*-129*	6.04	TTTATAGCtCatGCTATAAc	NRAMP-type Mn transporter / mntR
***Bradyrhizobium* sp. BTAi1**	Brad_2341	***mntH2***	*-91*	5.81	AATGTAGCagaaGCTAtATc	NRAMP-type Mn transporter
