# Supplementary material for: Computational Reconstruction of Iron- and Manganese-Responsive Transcriptional Networks in α-Proteobacteria
Source: PLoS Comput Biol. 2006 Dec 15;2(12):e163. doi: 10.1371/journal.pcbi.0020163 (PMC1698941; doi:10.1371/journal.pcbi.0020163)
Supplement: Supplementary file 8 [file pcbi.0020163.st006.pdf]

Suppl. Table 6. The Fur regulon in alpha-proteobacteria

Locus ID	Operon	Position	Score	Fur-box	Function
Caulobacter crescentus					
CC2928	*OMP1*	-32	5.85	ATTGCGACgCACTCGCAAT	TonB-dependent receptor
CC0028	*OMP2-piuC*	149	5.33	ATTGCGACGGCGcCGCAAc	TonB-dependent receptor, iron uptake factor piuC
CC0139	*OMP3*	-97	5.42	ATTGCaAGTCAGTCGCAtc	iron(III) dicitrate receptor fecA
CC2194	*OMP4*	-44	5.10	gTTGCGAGcCAtTCGCAtg	TonB-dependent receptor
CC2367		-130	5.05	tTTGCGAtTGACTCGCAAT	hypothetical protein
		-96	4.82	cTTGCGAagCAtTCtCAAg	
CC3263	*bfd-bfr*	-375	4.82	gaTGaGAaTGACaCtCAAT	bacterioferritin
CC0155		-31	4.45	ATTGaGAacCAtTCtCAAa	COG3205:short transmembrane protein
CC0711	*feoAB*	-144	4.05	AcTGatAaTCgtTCGCAAT	Fe2+ transport system
CC3059	*piuB*	-63	4.23	ATTGCaAtTCgtTCtCAAg	iron-uptake factor
Zymomonas mobilis					
ZMO0795	*OMP1*	-136	5.99	ATTGAaAATCAaTATCAAT	probable iron transport receptor
ZMO1631	*OMP2*	-74	5.88	ATTGAcAATCAaTATCAAT	TonB-dependent receptor
ZMO0318		-125	5.57	AgTGATAtTGATTtTCAAa	dehydrogenase/reductase family
ZMO1851	*nifF*	-89	5.48	ATTGATAtTGATaATCAtT	flavodoxin
ZMO0188	*OMP3*	-209	5.36	taTGATAtTGATTtgCAAT	ferric-pseudobactin receptor
ZMO1495		-132	5.24	ATTGATAtTGATTATtAtT	conserved hypothetical protein
ZMO1822	*OMP4*	-79	5.21	tgTGATAtTGATTcTCAtT	TonB-dependent receptor
ZMO0022		-237	5.17	AaTGAaAATCtTTATCAtT	Fe-S oxidoreductase
ZMO1860		-159	4.97	tTTGATAtTGATTATtAcT	nodulin 21 like protein
ZMO1540	*feoAB*	-159	4.94	ATTGATAAgCAgTtgCAtT	Fe2+ transport system
ZMO1753	*fpr*	-274	4.79	ATTGAgAtTtATTcgCAAa	ferredoxin-NADP reductase
ZMO1263	*adhA*	-240	4.78	tTTGcaAATGATTtTtAtT	alcohol dehydrogenase I
ZMO1410	*bfr*	-370	4.78	ATTGATAcTtgTTATCAtT	bacterioferritin
ZMO0031	*OMP5*	-233	4.59	tTcGATAtTCcTatTCAAT	TonB-dependent receptor
Gluconobacter oxydans					
GOX0536	*OMP1*	-145	6.22	AATGAGAATCATTtgCATT	Hydroxamate-type ferrisiderophore receptor
GOX0532	*exbBD-tonB*	-59	6.04	AgTGAGAATGAcTCTCATT	components for TonB-dependent iron transporters
GOX1246	*OMP2*	-100	5.89	AATGAGAATCtTTCgCAaT	TonB-dependent receptor
		-67	5.45	AtTGAGAATtAgTCgCATT	
GOX0673	*feoAB*	-106	5.79	gATGcaAATCATTCTCAcT	Fe2+ transport system
GOX0641	*OMP3*	-58	5.62	gtTGAGAATaATTaTCATT	Hydroxamate-type ferrisiderophore receptor
GOX1417	*OMP4*	-60	5.28	gtTGcGAATaAcTCgCATT	Ferrichrome receptor FcuA
Erythrobacter litoralis					
ELI2992	*feoAB*	-46	5.75	ATTGaGAATGAgTTGCAAT	Fe2+ transport system

ELI2950	*OMP1*	-65	5.59	ATTGCGAcTtATTCtCAAT	TonB-dependent receptor
		-31	4.55	ATcGaGAATCgTTCGCAAg	
ELI2144	*OMP2*	-61	5.17	ATTGCGAATacgTCtCAAT	outer membrane receptor for Fe3+-dicitrate
ELI1365		-126	5.06	gTTGCAAcTCATTCtCAtT	no hits, small CDS
ELI2069	*hemA*	-71	4.95	gaTGCGAATCATTCGCAAa	haem biosynthesis
ELI0967	COG3182	-46	4.9	gTTGCAAgTGATTatCAtT	uncharacterized iron-regulated membrane protein
ELI2935	*OMP3*	-243	4.8	AaaGCAAgTCAgTTtCAAT	TonB-dependent receptor
ELI1371		-30	4.78	ATcGCGAcTGcTTCGCgAT	hypothetical protein
ELI0014	*OMP4*	-235	4.71	ATTGCtgATCtTTaGCAAa	TonB-dependent receptor
ELI0267	*exbBD-tonB*	-8	4.57	cTcGCAAATGATTatCgAa	components for TonB-dependent iron transporters
ELI2812	*bfd-bfr*	-31	4.29	cTTGCGAATCAcTCGCAgg	bacterioferritin
Novosphingobium aromaticivorans					
Saro02000513	*OMP1*	-34	5.08	AgTGCGAtTGAgTCGCAaT	TonB-dependent receptor
		-6	4.27	ctTGCGAATGATTatCgTg	
Saro02001528	*OMP2-fegB*	-82	4.89	AATGCGAATGATTCttAgT	TonB-dependent receptor, iron-uptake factor
		-53	4.31	ctTGaGAAcGAcTCGCAcT	
Saro02002319	*feoAB*	-46	4.89	AtTGCGAAcGAcTTGCAaT	Fe2+ transport system
Saro02003622	*OMP3*	-153	4.53	AATGCAAtTGggcaGCATT	TonB-dependent receptor
Saro02003557	*OMP4*	-228	4.28	tAcGCAAATGATTTtCgca	TonB-dependent receptor
Rhodospirillum rubrum ATCC11170					
Rru_A2597	*ftr2-chpA2-COG2837*	-63	5.61	AtTGATAATTAaTCTCATT	High-affinity Fe2+ transport component and permease, peroxidase
Rru_A0841	*araX3*	-180	5.41	AAcGATAATTAaTATCATT	siderophore uptake AraC-type regulator
Rru_A2799	*Rru_A2799-2796*	-199	5.28	AATGAaAgTCATTCgCAaT	COG0581: ABC-type phosphate transport system
Rru_A2763		-96	5.19	AATGAGAgTGATTCTCATg	Putative diguanylate cyclase/phosphodiesterase
Rru_A0880	*OMP2*	-170	5.07	AAcGAGAcTTATTAgCAaT	TonB-dependent receptor
Rru_A2321		-59	5.07	AATGcTAtTGAaTATCgaT	chemotaxis protein
Rru_A1054	*amb3711*	-119	4.97	AAcGATtATCAaTAgCATT	hypothetical protein
Rru_A0879	*araX2*	-99	4.94	AtTGcaAATGAcTtgCAaT	siderophore uptake AraC-type regulator
Rru_A1438	*fecIR1 -OMP1*	-59	4.85	ttTGcGAATAATTCTCAaa	iron transport sigma factor and sensor, TonB-dependent receptor
Rru_A0845	*OMP7-fegB*	-131	4.85	tATGATAATAAgaAgCATT	TonB-dependent receptor, iron-uptake factor
Rru_A2196	*bfd-bfr*	-74	4.74	AATGcGAtTGAcTtgCAaa	COG2906: Bacterioferritin-associated ferredoxin, bacterioferritin
Rru_A1456	*araX1*	-114	4.61	cATGAGAcgCATTtTCATT	siderophore uptake AraC-type regulator
Rru_A2155	*exbBD-tonB*	-196	4.48	AtTaATAgTCAaTtgCATT	iron transport components
Rru_A0769	*fbpABC*	-77	4.45	tcTGcGttTCATTCTCAaT	ferric cations ABC transporter
Rru_A2372	*fecIR4 -OMP4*	18	4.43	AATGcGAAgCgTTtgCATT	iron transport sigma factor and sensor, TonB-dependent receptor
Rru_A2397	*fecIR2*	-102	4.4	AATGATAgTCgTTCgCAaa	iron transport sigma factor and sensor
Rru_A2809	*ftr-chpA*	-60	4.35	AATGAaAATGcaTggCtTT	High-affinity Fe2+ transport component and permease
Rru_A2395	*OMP5-piuB*	-52	4.34	cATGcGAAcAAgTCgCATT	TonB-dependent receptor, iron-uptake factor
Magnetospirillum magneticum AMB-1					

amb1811		-35	5.99	AtTGCGAGTCAtTCGCATT	hypothetical protein, predicted transcriptional regulator
amb3711		-39	5.99	AtTGCGAGTAAtTCGCATT	hypothetical protein, ortholog of Rrub02001888
amb1662	*~irr - bfr1-bfr2*	-33	5.58	gtTGCGAGTAAaTCGCAaT	regulator, bacterioferritin
amb1681	*ftr2*	-70	5.42	AATGCGAGcTAtTtGCAaT	High-affinity Fe2+ permease
amb1008		-38	5.22	gATGCGAtTTgtTCGCAaT	unknown hypothetical short ORF
amb3013	*cycC -nirB-nrfAB*	-154	5.22	gtTGCGAtcGAtTCGCATT	cytochrome, nitrite reductase
amb0918		-175	5.18	gATGCaAGTTAaTCGCAac	CheY-like receiver
amb4411	*chpA1-oprB1- COG0348*	-2	5.88	AATGCGAGTCACTtGCATT	protein involved in high-affinity Fe2+ transport, porin, polyferredoxin
amb0954	*mms6-mms7-mms13*	-174	4.94	cATGCaAaTCACTCtCAac	bacterial magnetic particle formation proteins
amb0955	*amb0955- mms6*	-91	4.94	gtTGaGAGTGAtTtGCATg	bacterial magnetic particle formation proteins
amb1424	*amb1424- bfd*	-41	4.83	gATGCGAGTCAtTatCATg	?, COG2906: Bacterioferritin-associated ferredoxin
amb0936	*amb0936-ftr1- COG0348 -chpA2*	-41	4.78	AATtCGAGTCAtTaGCAaa	High-affinity Fe2+ transport component and permease, ferredoxin
amb2978	*sodC*	-146	4.72	ctTGCGcGTGAaTCGCATc	Cu/Zn superoxide dismutase
amb1022	*oprB2- feoAB2*	-42	4.69	gATGCGAGcCAgTCGCAcT	porin, Fe2+ transport system
amb0940	*chpA3-oprB3*	-2	4.31	AATGCGtcTCACTCtCgTg	protein involved in high-affinity Fe2+ transport
amb2732	*feoAB1*	-33	4.27	gATGCGAtgCgCTtGCATc	Fe2+ transport system
amb3546	*X- fepA-exbB-tonB- tolQ -exbD1-ex*	-30	4.2	gtTGatAaTCAtTCGCAgc	TonB-dependent receptor, iron transport components
Expression data	***Iron up-regulated genes (Suzuki et al., 2006)***				
Magnetospirillum magnetotacticum MS-1					
Magn03009213	*chpA*	-48	5.83	AtTGCGAGTCACTtGCATT	protein involved in high-affinity Fe2+ transport
Magn03009755	*amb1811*	-80	5.84	AtTGCGAtTCACTCGCATT	hypothetical protein, predicted transcriptional regulator
Magn03007091	*amb1008*	-38	5.45	gATGCGAGTTgtTCGCAaT	unknown hypothetical short ORF
Magn03010317	*ftr2*	-77	5.32	AATGCaAtTCgCTCGCATT	High-affinity Fe2+ transport permease
Magn03010302		-90	5.02	ttTGCtAtTGACTCGCAaT	Methyl-accepting chemotaxis protein
Magn03011341	*Magn03011341-Magn03011342*	-129	5.06	ttTGCGAtcAAtTCGCATT	Predicted Fe-S oxidoreductases
Magn03007981	*mms6-mms7-mms13*	-174	4.94	cATGCaAaTCACTCtCAac	bacterial magnetic particle formation proteins
Magn03007982	*amb0955- mms6*	-91	4.94	gtTGaGAGTGAtTtGCATg	bacterial magnetic particle formation proteins
Magn03009364	*oprB-feoAB2*	-230	4.79	gATGCGAGcTAaTCGCAcT	porin, Fe2+ transport system
Magn03007972	*amb0936-ftr1*	-63	4.78	AATtCGAGTCAtTaGCAaa	High-affinity Fe2+ transport component and permease
Magn03007235		-97	4.75	gtTGCGAtTTAtTtGCAcT	Methyl-accepting chemotaxis protein
Magn03006859	*mxcB*	-411	4.43	AcTtCGAaTAAaTCtCAaT	siderophore-interacting protein
Magn03008031	*bfd*	-51	4.35	gtTGCGAcTGAtTatCATa	COG2906: Bacterioferritin-associated ferredoxin
Magn03007374	*feoAB*	-7	4.27	gATGCGAtgCgCTtGCATc	Fe2+ transport system
***Sphinopyxis alaskensis* RB2256**					
Sala_2282	*OMP1*	-48	4.78	tTTGCGAATGtgTCGCAcT	TonB-dependent siderophore receptor
		-8	4.84	gTTGatAATGAaTCGCAAa	
Sala_2061	*OMP2*	-63	5.19	AaTGCaAcTaATTCGCAtT	TonB-dependent siderophore receptor
Sala_1475	*OMP3*	201	5	gTTGCaAtTCgcTCGCAtT	TonB-dependent siderophore receptor
Sala_1279	*feoAB*	-261	5.04	ATTGatAATCgcTCGCAAa	Fe2+ transport system

Sala_1595	*hemA*	-118	5.52	AcTGCGAATCgTTCGCAcT	haem biosynthesis
Sala_1122	*sufS* -*Sala_1121*	-35	5.84	tTTGCGAATtgTTCGCAAT	Fe-S cluster assembly, ferredoxin
Sala_1236	*iscR-sufBCDSA*	-35	5.16	gTTaaGAATCgTTCGCAAc	Fe-S cluster assembly
Sala_0415	*bfr*	-354	5.19	AcTGCGAAcCAgTCGCAtc	bacterioferritin
		-329	4.85	gTTGCGAATCtgTCGCAcT	
Sala_1982	*fpr*	-189	5.01	ATTGCaAATGAgTtGCAcT	ferredoxin-NADP reductase
***Parvularcula bermudensis* HTCC2503**					
PB2503_04802	*sdh*	-115	5.94	tTTGCGAATGATTatCAAT	succinate dehydrogenase
PB2503_07374	*OMP1*	-32	5.99	ATTGaGAATGATTatCAAT	TonB-dependent siderophore receptor
PB2503_08524	*OMP2*	-34	5.77	tTTGCaAgTCATTCGCAAT	TonB-dependent siderophore receptor
PB2503_13539	*fbpCA*	-34	5.86	ATTGaGAActATTCGCAAa	ferric cations ABC transporter
PB2503_13544	*fbpB*	-71	5.86	tTTGCGAATagTTCtCAAT	ferric cations ABC transporter
PB2503_00005	*hmuR*	-32	5.87	ATTGaGAgTCATTCGCAAg	TonB-dependent haemoglobin/transferrin/lactoferrin receptor
PB2503_00035	*hmuTUV*	-99	5.81	AaTGCGAATtATTCGCAta	hemin ABC transporter
PB2503_13529	*piuC*	-47	<u>5.07</u>	tTTGCaAATGccTCGCAAa	COG3128 Uncharacterized iron-regulated protein
***Oceanicaulis alexandrii* HTCC2633**					
OA2633_01936	*hemA*	-144	4.46	ttTGAGAAgCgTTgTCAag	haem biosynthesis
OA2633_02911	*nuoABCDEFGHIJLMN*	-220	4.77	ttTGAGAggCATTCgCAaa	NADH ubiquinone oxidoreductase
OA2633_04511	*OMP1-X-piuC2*	-82	4.81	tCTGcaAtTCATTCTCATT	TonB-dependent siderophore receptor
OA2633_05321	*OMP2*	-38	4.54	AAcGAaAAgCATTCTCATc	TonB-dependent siderophore receptor
OA2633_11590	*piuC*	-26	4.73	ACTGcaAATCAcTCgCAGg	COG3128 Uncharacterized iron-regulated protein
OA2633_12420	*bfr*	-103	4.75	tCTGcaAcTCAgTtTCAGT	bacterioferritin
OA2633_12430	*bfd*	-76	4.75	AtTGcGAATGATTtTCgGa	COG2906 Bacterioferritin-associated ferredoxin
OA2633_12925	*OMP3*	-537	4.79	tATGAGAATGATcCgCATT	TonB-dependent siderophore receptor
OA2633_08069	*OMP4-piuB*	-46	4.38	AtTGAtAATCAcTCTCAaa	TonB-dependent siderophore receptor, iron-uptake factor
OA2633_08249	*hugA*	-118	4.51	AgTGcGAAaGATTCTCAcT	TonB-dependent haemoglobin/transferrin/lactoferrin receptor
		-64	4.5	AtTGAGAActATTCgCAaT	
OA2633_09259	*sdh*	-133	4.88	ACTGAGAAcCgTTtgCAaa	succinate dehydrogenase
OA2633_15080	*fbpABC*	-88	4.41	ctTGcGAgaCgTTCTCAGa	ferric cations ABC transporter
OA2633_10854		-29	4.84	ttTGcGAATGgTTCgCATg	COG3205:short transmembrane protein
***Pelagibacter ubique* HTCC1002**					
PU1002_00070	*fbpABC*	-70	5.13	AtTGatAaTCAtTCGCAaT	ferric cations ABC transporter

<u>Color code for functional roles of genes:</u>

Genes involved in iron uptake

Genes involved in iron storage

Genes involved in iron utilization pathways (synthesis of Fe-S, heme; iron-containing enzymes)

Transcription factors (rirA, araX, fecIR, irr)

Genes with other or uncertain functional roles
