# Supplementary material for: Computational Reconstruction of Iron- and Manganese-Responsive Transcriptional Networks in α-Proteobacteria
Source: PLoS Comput Biol. 2006 Dec 15;2(12):e163. doi: 10.1371/journal.pcbi.0020163 (PMC1698941; doi:10.1371/journal.pcbi.0020163)
Supplement: Supplementary file 9 [file pcbi.0020163.st007.pdf]

Suppl. Table S7. The IscR regulon in alpha-proteobacteria

Organism	Locus ID	Operon	Positi	Score	Site
Consensus 1:					CTTGACgrwwwnnnTCrrr
Silicibacter sp.	*TM1040_1240*	*iscR-sufSBCD*	-138	5.72	CTTGACGAATTttaTCcGG
Silicibacter pomeroyi	*SPO2025*	*iscR-sufSBCD*	-140	6.09	CTTGACGAATTCcCTCGGG
Rhodobacter sphaeroides	*RSP_0443*	*iscR-sufSBCD*	-120	5.13	CTTGACaAAAaCGcTCgga
Rhodobacter capsulatus	*001_0477*	*iscR-sufSBCD*	-151	4.01	CTTGACCGtcTCGcTCAtG
Roseobacter sp. MED193	*MED193_04321*	*iscR-sufSBCD*	-143	5.58	CTTGACGAATTCcaTCaGc
Roseovarius sp.217	*ROS217_20542*	*iscR-sufSBCD*	-119	6.09	CTTGACGAcTTCGCTCGGG
Roseovarius nubinhibens ISM	*ISM_16015*	*iscR-sufSBCD*	-148	4.55	CTTGACGgAgcgGCcaGGt
Loktanella vestfoldensis SKA53	*SKA53_05183*	*iscR-sufSBCD*	-130	5.36	CTTGACGAAaaCtaTCaGG
Sulfitobacter sp. EE-36	*EE36_14302*	*iscR-sufSBCD*	-137	5.69	gTTGACGAATTCGCTaGGG
Oceanicola batsensis HTCC2597	*OB2597_03589*	*iscR-sufSBCD*	-133	5.12	CTTGACGAcTTtGgTCaaG
Rhodobacterales bacterium HTCC2654	*RB2654_04009*	*iscR-sufSBCD*	-144	4.52	CTTGACtggcTaGacCGGG
Pelagibacter ubique **HTCC1002**	*PU1002_02686*	*iscR-sufBCDS-fss*	-51	4.71	CTTGACtAATTtagTCaGG
Rhodospirillum rubrum	*Rrub02002541*	*cysE-iscR1-sufS1*	-163	4.85	CTTGACcGATTTTcTCGGA
Magnetospirillum magneticum	*amb3031*	*cysE-iscR-sufS*	-113	5.09	CTTGACtGGCCTTGTCGGA
Parvularcula bermudensis **HTCC2503**	*PB2503_09884*	*iscR-sufSBCD*	-139	3.66	gTTGACcggAaagGTCgga
Rickettsia conorii	*RC0732*	*iscRSSUA*	-42	6.35	ATTGTAGAtATTAAACAAT
Rickettsia sibirica	*rsib_orf.1360*	*iscRSSUA*	-75	6.35	ATTGTAGAtATTAAACAAT
Rickettsia prowazekii	*RP488*	*iscRSSUA*	-43	5.95	ATTGTAGAcATaAAACAAT
Consensus 2:					tcgGAcyrATtnrGTCynr
Erythrobacter litoralis	*ELI0458*	*iscR-sufB-..*	-38	5.46	TCGGAacGATTTgGTCCGA
Erythrobacter **sp. NAP1**	*NAP1_13233*	*iscR-sufB-..*	-59	5.72	TCcGACTGATTCAGTCCGA
Novosphingobium aromaticivorans	*Saro02000260*	*iscR-sufB-..*	-64	5.63	cCGGAgTGATTCgGTCCGA
Sphinopyxis alaskensis **RB2256**	*Sala_1236*	*iscR-sufB-..*	-53	4.74	gtGGAgTAATTTAcTCtag
Zymomonas mobilis	*ZMO0422*	*iscR-sufB-..*	-109	5	TCaGACaAATTTAGTCttg
Gluconobacter oxydans	*GOX0099*	*iscR-sufB-..*	-47	4.29	caGGACTtAgcaAGTCttg
	GOX0777	*fssA*	-7	4.35	TCGGACcAATgCAGTCCcg
